# An in vitro vesicle formation assay reveals cargo clients and factors that mediate vesicular trafficking

**DOI:** 10.1073/pnas.2101287118

**Published:** 2021-08-25

**Authors:** Yan Huang, Haidi Yin, Baiying Li, Qian Wu, Yang Liu, Kristina Poljak, Julija Maldutyte, Xiao Tang, Mo Wang, Zhixiao Wu, Elizabeth A. Miller, Liwen Jiang, Zhong-Ping Yao, Yusong Guo

**Affiliations:** ^a^Division of Life Science and State Key Laboratory of Molecular Neuroscience, The Hong Kong University of Science and Technology, Hong Kong, China;; ^b^State Key Laboratory of Chemical Biology and Drug Discovery, Research Institute for Future Food, Department of Applied Biology and Chemical Technology, The Hong Kong Polytechnic University, Hung Hom, Kowloon, Hong Kong Special Administrative Region, China;; ^c^State Key Laboratory of Chinese Medicine and Molecular Pharmacology (Incubation), Shenzhen Key Laboratory of Food Biological Safety Control, Shenzhen Research Institute of Hong Kong Polytechnic University, Shenzhen 518057, China;; ^d^School of Life Sciences, Centre for Cell and Developmental Biology, State Key Laboratory of Agrobiotechnology, The Chinese University of Hong Kong, Shatin, New Territories, Hong Kong, China;; ^e^Cell Biology Division, Medical Research Council Laboratory of Molecular Biology, CB2 0QH, Cambridge, United Kingdom;; ^f^Shenzhen Research Institute, The Hong Kong University of Science and Technology, 518057 Shenzhen, China;; ^g^Hong Kong Branch of Southern Marine Science and Engineering Guangdong Laboratory (Guangzhou), The Hong Kong University of Science and Technology, Clear Water Bay, Hong Kong, China

**Keywords:** cargo sorting, secretory pathway, intracellular protein transport, COPII, cargo receptor

## Abstract

The fidelity of protein transport in the secretory pathway relies on the accurate sorting of proteins to their correct destinations. To deepen our understanding of the underlying molecular mechanisms, it is important to develop a robust approach to systematically reveal cargo proteins that depend on specific sorting machinery to be enriched into transport vesicles. Here, we used an in vitro assay that reconstitutes packaging of human cargo proteins into vesicles to quantify cargo capture. Quantitative mass spectrometry (MS) analyses of the isolated vesicles revealed cytosolic proteins that are associated with vesicle membranes in a GTP-dependent manner. We found that two of them, FAM84B (also known as LRAT domain containing 2 or LRATD2) and PRRC1, contain proline-rich domains and regulate anterograde trafficking. Further analyses revealed that PRRC1 is recruited to endoplasmic reticulum (ER) exit sites, interacts with the inner COPII coat, and its absence increases membrane association of COPII. In addition, we uncovered cargo proteins that depend on GTP hydrolysis to be captured into vesicles. Comparing control cells with cells depleted of the cargo receptors, SURF4 or ERGIC53, we revealed specific clients of each of these two export adaptors. Our results indicate that the vesicle formation assay in combination with quantitative MS analysis is a robust and powerful tool to uncover novel factors that mediate vesicular trafficking and to uncover cargo clients of specific cellular factors.

## Significance

Protein sorting in the secretory pathway is a fundamentally important cellular process, but the clients of a specific cargo sorting machinery remains largely underinvestigated. Here, utilizing a vesicle formation assay to profile proteins associated with vesicles, we identified cytosolic proteins that are associated with vesicle membranes in a GTP-dependent manner or that interact with GTP-bound Sar1A. We found that two of them, FAM84B and PRRC1, regulate anterograde trafficking. Moreover, we revealed specific clients of two export adaptors, SURF4 and ERGIC53. These analyses demonstrate that our approach is powerful to identify factors that regulate vesicular trafficking and to uncover clients of specific cargo receptors, providing a robust method to reveal insights into the secretory pathway.

The eukaryotic secretory pathway plays important roles in delivering a variety of newly synthesized proteins to their specific resident compartments. The fidelity of protein transport in the secretory pathway depends on accurate sorting of specific cargo proteins into transport vesicles. Defects in cargo sorting cause protein mistargeting and induce defects in establishing cell polarity, immunity, as well as other physiological processes (1).

A variety of cytosolic proteins are recruited to the membrane and play important roles in the protein sorting process. These cytosolic proteins include small GTPases of the Arf family and cargo adaptors (1, 2). The Arf family GTPases cycle between a GDP-bound cytosolic state and a GTP-bound state. Upon GTP binding, Arf proteins undergo conformational changes in which the N-terminal amphipathic helix is exposed to bind membranes and the switch domains change their conformation to recruit various cytosolic cargo adaptors. Once recruited onto the membranes, these cargo adaptors recognize sorting motifs on the cargo proteins. This recognition step is important for efficiently capturing cargo proteins into vesicles.

The Arf family protein, Sar1, regulates packaging of cargo proteins into vesicles at the endoplasmic reticulum (ER). GTP-bound Sar1 mediates membrane recruitment of the coat protein complex II (COPII) to capture cargo proteins (2). Soluble cargo proteins in the lumen of the ER cannot be directly recognized by COPII coat and such proteins are thought to be linked to the cargo sorting machinery on the cytosolic side by transmembrane cargo receptors. One cargo receptor in mammalian cells, ERGIC53, is a mannose lectin and functions in capturing specific N-linked glycoproteins in the lumen of the ER (3). ERGIC53 regulates ER export of blood coagulation factors V and VIII, a cathepsin-Z–related protein, and alpha1-antittrypsin (4–7). Another cargo receptor, SURF4, binds amino-terminal tripeptide motifs of soluble cargo proteins and regulates ER export of soluble cargo proteins, including the yolk protein VIT-2 in *Caenorhabditis elegans* (8), and PCSK9 and apolipoprotein B in mammalian cells (9–11).

Although significant progress has been made in understanding the general steps of cargo sorting, the spectrum of cargo clients of a specific Arf family member, cargo adaptor, or cargo receptor remains largely underinvestigated. To deepen our understanding of protein sorting in the secretory pathway, it is important to develop a robust approach to systematically reveal cargo proteins that depend on a specific factor to be efficiently packaged into vesicles. Revealing this will provide significant insight into the functions and the specificity of cargo sorting. Since distinct cytosolic proteins are recruited to membranes by different GTP-bound Arf family proteins, systematic approaches are needed to characterize budding events associated with a specific GTP-bound Arf family protein.

A cellular imaging approach, pairing analysis of cargo receptors (PAIRS), has been utilized to identify the spectrum of cargo proteins that depend on a specific cargo receptor for ER export in yeast. This analysis focused on around 150 cargo molecules labeled with fluorescent tags (12). An in vitro assay that reconstitutes packaging of cargo proteins into vesicles has been used to reveal protein profiles of vesicles budded with purified COPII or COPI proteins (13). However, this analysis did not identify any non-ER resident transmembrane proteins or secretory proteins (13). This is possibly due to an unappreciated requirement for other cytosolic factors in addition to the COP coats. Affinity chromatography has been utilized to reveal cytosolic proteins that specifically interact with GTP-bound Arf or Rab proteins (14–16). In this approach, the membranes are disrupted, which might preclude identification of membrane-associated effectors. Thus, it is important to develop additional approaches to reveal novel cytosolic proteins that associate with GTP-bound Arf proteins on membranes.

Here, we used an in vitro assay to reconstitute packaging of cargo proteins into transport vesicles utilizing rat liver cytosol (RLC) as a source of cytosolic proteins. Analysis of vesicle fractions by quantitative mass spectrometry (MS) revealed cytosolic proteins that are associated with vesicles dependent on GTP or GTP-bound Sar1A, and that regulate protein trafficking. One of the identified proteins, PRRC1, regulates membrane association of the COPII coat and facilitates ER-to-Golgi trafficking. We also revealed cargo proteins that depend on specific cargo receptors, ERGIC53 or SURF4, to be efficiently packaged into vesicles. Our study indicates that the vesicle formation assay is a robust tool to reveal functional roles of specific factors in protein sorting, and to uncover novel factors that regulate vesicular trafficking in the secretory pathway.

## Results

### An In Vitro Reconstituted Vesicle Formation Assay for Proteomic Analysis.

**Fig. 1.**
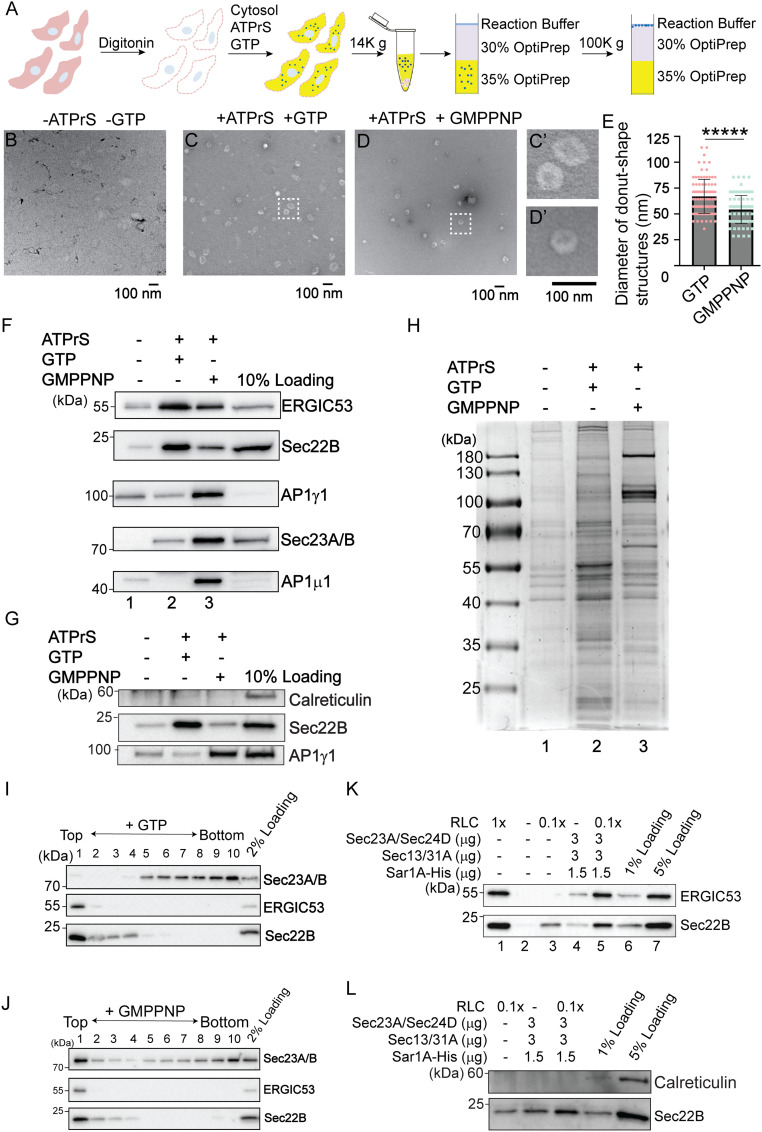
A large-scale in vitro vesicle formation assay for proteomic analysis. (*A*) Diagram demonstrating the experimental procedures for the vesicle formation assay. (*B–D*) Visualization of the morphology of the buoyant membrane structures formed in the budding reaction. The buoyant membranes were isolated by density gradient flotation and analyzed by negative staining TEM. (*C′* and *D′*) The magnified views of the indicted areas in *C* and *D*. (Scale bar, 100 nm.) (*E*) Quantification of the diameter of donut shape structures from three biological repeats (mean ± SD *****P < 0.00001). (*F–H*) The vesicle formation assay was performed using the indicated reagents. Vesicle fractions were analyzed by immunoblot (*F* and *G*) or Coomassie blue staining (*H*). ATPrS: ATP regeneration system. (*I* and *J*) The vesicle formation assay was performed in the presence of GTP (*I*) or GMPPNP (*J*). The vesicle fractions were evaluated by density gradient flotation. (*K* and *L*) The vesicle formation assay was performed using the indicated reagents. The vesicle fraction was analyzed by immunoblot using the indicated antibodies. Data shown in *F*, *G*, and *K* are representative example of three biological repeats.

An in vitro vesicle formation assay to reconstitute packaging of cargo proteins into vesicles from mammalian cells has been well established (17–21). We sought to perform this assay in HEK293T cells on a large scale and then perform proteomic analysis on the isolated vesicles. The general procedures of the vesicle formation assay are shown in Fig. 1*A*. Briefly, HEK293T cells were permeabilized by digitonin, after which the semi-intact cells were washed with buffer to remove cytosolic proteins. Washed semi-intact cells were then incubated at 30 °C with RLC, GTP, and an ATP regeneration system (ATPrS). The small vesicles released during this incubation were separated from the heavy donor membranes by medium speed centrifugation. The supernatant containing the vesicle fraction was adjusted to 35% Opti-Prep and overlaid with layers of 30% Opti-Prep and the reaction buffer. The samples were then centrifuged to float the vesicles away from cytosolic proteins that are not associated with membranes. Two control experiments were performed: one performed in the absence of GTP and ATPrS and the other performed in the presence of a nonhydrolyzable analog of GTP, GMPPNP.

We performed negative stain electron microscopy to visualize the morphology of the buoyant membrane structures produced in the vesicle budding reaction. We detected numerous small membrane structures with an average diameter of 67 nm (Fig. 1 *C* and *E*). When we performed the vesicle budding reaction in the absence of GTP and ATPrS or in the presence of GMPPNP, the number of vesicles was greatly reduced (Fig. 1 *B* and *D*). The average diameter of the membrane structures produced in the presence of GMPPNP was significantly reduced to 54 nm (Fig. 1*E*, magnified views in Fig. 1 *C′* and *D′*). These analyses are consistent with the slowly sedimenting membranes in the budding reaction representing transport vesicles rather than fragments of the ER or Golgi.

The buoyant membranes were analyzed by immunoblotting with antibodies against standard cargo proteins in COPII vesicles, Sec22B (a tSNARE), and ERGIC53. Capture of ERGIC53 and Sec22B into the vesicle fraction was enhanced by the ATPrS and GTP (Fig. 1*F*, compare lanes 1 and 2) and reduced in the presence of GMPPNP (Fig. 1*F*, compare lanes 2 and 3), suggesting that GTP hydrolysis is important for efficient packaging of cargo proteins into transport vesicles. In contrast, vesicle coat proteins, including the γ- and μ-subunits of the adaptor complex 1 (AP1γ1 and AP1μ1) and the inner COPII subunit Sec23A/B, were more robustly associated with the vesicle fraction in the presence of GMPPNP (Fig. 1*F*, compare lanes 2 and 3). The ER resident protein, calreticulin, was not detected in the vesicle fraction in all of the experimental groups (Fig. 1*G*). These results confirm that GTP hydrolysis permits release of AP-1 and COPII from membranes (1, 2), and that this recycling is important to sustain efficient vesicle formation. We next analyzed the proteins in the buoyant vesicle fractions by sodium dodecyl sulphate-polyacrylamide gel electrophoresis (SDS-PAGE) and Coomassie blue staining (Fig. 1*H*), noting distinct protein compositions for the different reaction conditions. Again, the pattern of protein recovery is consistent with coat proteins stabilized in the presence of GMPPNP and more robust vesicle release in the context of ATPrS and GTP. Finally, we assessed the distribution of cargo and coat proteins throughout the OptiPrep gradient, finding Sec22B and ERGIC53 enriched in the top fraction (Fig. 1 *I* and *J*). We detected Sec23A/B in the floated fraction only when the vesicle formation assay was performed in the presence of GMPPNP (Fig. 1 *I* and *J*).

Since cytosol was used as the source of coat proteins in these experiments, multiple types of vesicles may be formed. Sec22B and ERGIC53 were packaged into the vesicle fraction with purified COPII, albeit with reduced efficiency compared to reactions with cytosol (Fig. 1*K*, compare lanes 1 and 4). Purified COPII proteins have previously been shown to promote vesicular release of ER-Golgi cargo proteins (17, 22). Consistently, when RLC at low concentration was supplemented with purified COPII, release of Sec22B and ERGIC53 into vesicles was enhanced (Fig. 1*K*, compare lane 5 with lanes 3 and 4), whereas calreticulin was not detected (Fig. 1*L*). These analyses indicate that some proteins in RLC work together with purified COPII to promote packaging of cargo proteins into vesicles. Therefore, we utilized cytosol prepared from rat liver to provide a source of cytosolic proteins in the vesicle formation assay for our subsequent quantitative analysis.

### Identification of Cytosolic Proteins Associated with Vesicles in a GTP-Dependent Manner.

**Fig. 2.**
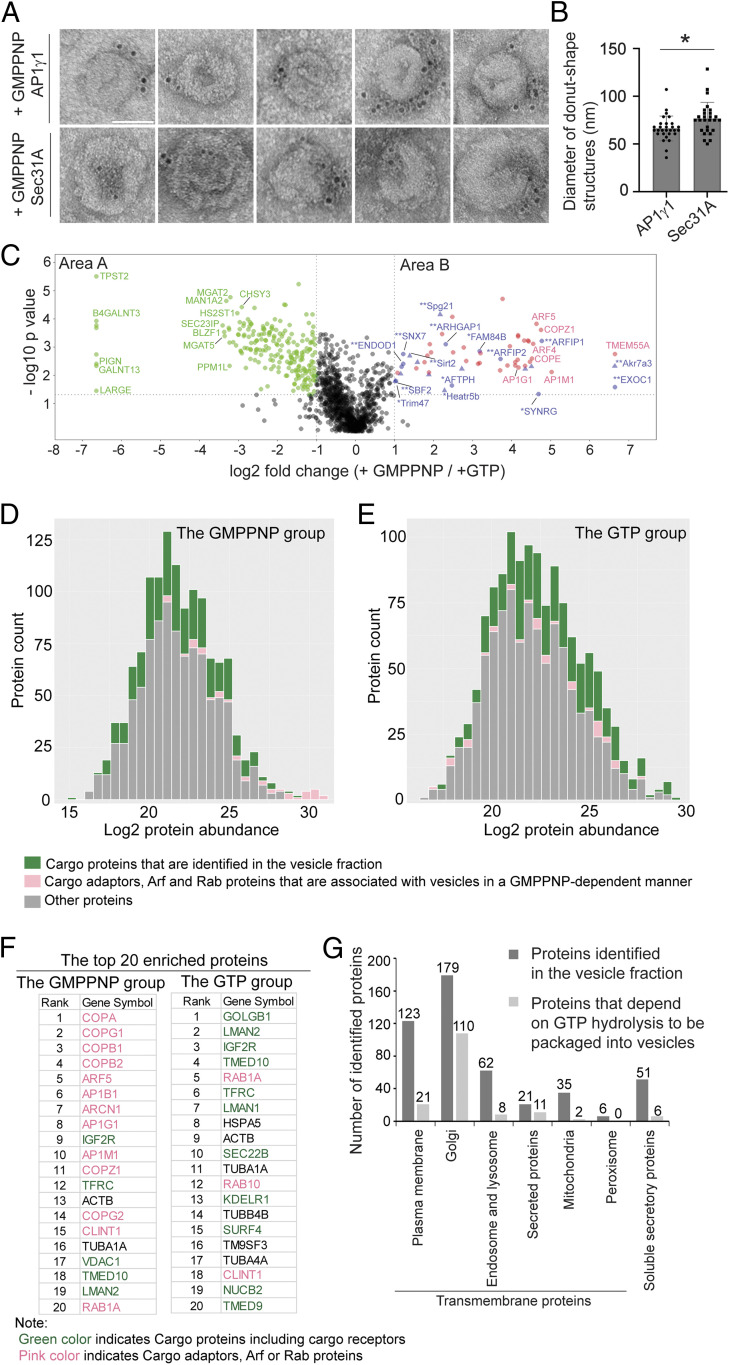
Identification of cytosolic proteins that are associated with vesicles in a GTP-dependent manner and cargo proteins that are packaged into vesicles in a GTP-hydrolysis–dependent manner. (*A*) Immunogold TEM was performed using AP1γ1 and Sec31A antibodies to label the donut shape structures produced in the presence of GMPPNP. (Scale bar, 50 nm.) (*B*) Quantification of the diameter of the donut shape structures labeled by antibodies against AP1γ1 and Sec31A (mean ± SD *P < 0.05, from two biological repeats). (*C*) The vesicle formation assay was performed in the presence of GTP or GMPPNP. The isolated vesicles in each experimental group were resuspended in RapiGest SF surfactant. The proteins in the vesicle fractions were trypsin digested and analyzed by label-free mass spectrometry. A total of 1,285 proteins were identified in both experimental groups. The log2 ratio of the abundance of each identified protein in the vesicles prepared in the presence of GMPPNP over that in the vesicles prepared in the presence of GTP was plotted on the *x* axis and the –log10 *P* value of the difference was plotted on the *y* axis. (*D* and *E*) Histogram of the log2 abundance of the human proteins identified in the vesicle fraction produced in the presence of GMPPNP (*D*) or GTP (*E*). (*F*) The list of the top 20 abundant human proteins in the GMPPNP group or in the GTP group. (*G*) Number of proteins categorized based on predictions from Uniprot.

Immunogold labeling experiments indicated that many of the vesicular structures produced in the presence of GMPPNP were labeled with antibodies against AP1γ1 or the outer COPII subunit, Sec31A (Fig. 2*A*). In contrast, we did not detect vesicular structures produced in the presence of GTP that were labeled by these antibodies. Interestingly, the average diameter of vesicular structures labeled by AP1γ1 was significantly lower than those labeled by Sec31A (66 nm vs. 77 nm, Fig. 2*B*), suggesting AP-1–coated vesicles are smaller than COPII-coated vesicles.

To gain a comprehensive view of cytosolic proteins that are associated with vesicle membranes in a GTP-dependent manner, we performed label-free quantitative mass spectrometry to compare protein profiles of the vesicle fractions in GTP vs. GMPPNP treatment conditions based on three biological repeats. A total of 1,285 proteins were identified and quantified, all of which had two or more unique peptides with a false discovery rate (FDR) of <0.01 and were successfully quantified in all of the three biological repeats (*SI Appendix*, Table S1, sheet 1). The fold changes of the identified proteins in the GMPPNP group compared with the GTP group were quantified. Based on protein abundance, a *P* value was calculated and plotted against the mean log2 fold changes. Proteins with a fold change of >2 and a *P* value of <0.05 are considered as significant hits. Through this approach, 54 proteins were identified as having more than twofold enrichment in the GMPPNP group over the GTP group (*P* < 0.05, Fig. 2*C*, area B; proteins identified using the protein sequence database of *Homo sapiens* are indicated in round shapes and additional proteins identified using the database of *Rattus norvegicus* are indicated in triangle shapes; *SI Appendix*, Table S1, sheet 2). In addition, a permutation-based FDR (*q* value) was calculated (23). Most identified hits (except two proteins) showed *q* value of <0.05 (*SI Appendix*, Table S1, sheet 2). A total of 36 proteins (67%) were known Arf family proteins, Rab proteins, and cargo adaptors (Fig. 2*C*, area B, marked in pink).

A stoichiometric analysis of the cargo proteins and cargo adaptors is shown in Fig. 2 *D* and *E*. Cargo adaptors, Arf, and Rab proteins, were significantly enriched in the GMPPNP group compared with the GTP group (*P* < 0.01) (Fig. 2*D*, marked in pink) and constituted 65% of the top 20 most abundant proteins (13 out of 20 proteins) in the GMPPNP group (Fig. 2*F*, marked in pink). We identified several cytosolic proteins in addition to Arf, Rab, and known cargo adaptor proteins that are associated with vesicles in a GTP-dependent manner (Fig. 2*C*, area B, marked in blue and *SI Appendix*, Table S1, sheet 4). We hypothesize that these proteins may be cargo adaptors or proteins associated with vesicle coats. Proteins highlighted with double asterisks are those first predicted by the present study to be associated with coated vesicles. Proteins highlighted with a single asterisk were predicted to be associated with clathrin-coated vesicles in previous studies (24–26). The cellular functions of the majority of these proteins are unclear.

### Identification of Cargo Proteins Enriched in Vesicles in a GTP-Hydrolysis–Dependent Manner.

Next, we characterized cargo proteins that are packaged into vesicles in the vesicle formation assay. We defined cargo proteins as soluble secretory proteins or transmembrane proteins that are localized at the Golgi, endosomes, lysosomes, or plasma membrane. For simplification, we classified transmembrane cargo receptors as cargo proteins. Cargo proteins, contrary to cargo adaptor proteins, were enriched in the GTP group (*P* < 0.01) (Fig. 2*E*, marked in green) and constituted 55% of the top 20 most abundant proteins (11 out of 20 proteins) in the GTP group (Fig. 2*F*, marked in green). Further analysis indicates that 4% (51 proteins) of the proteins identified in the vesicle fraction were predicted by Uniprot annotation to be soluble secretory cargo proteins (Fig. 2*G* and *SI Appendix*, Table S1, sheet 5). A total of 37% (482 proteins, *SI Appendix*, Table S1, sheet 5) of the proteins identified in the vesicle fraction were predicted to be transmembrane proteins. Among the predicted transmembrane proteins, 179 are predicted to show Golgi localization, 21 proteins can be secreted presumably in extracellular vesicles, 123 proteins are predicted to show plasma membrane localization, and 62 proteins show endosomal and lysosomal localization (Fig. 2*G* and *SI Appendix*, Table S1, sheet 5). We also detected several transmembrane proteins that are predicted to be located at the mitochondria or peroxisome in the vesicle fraction (Fig. 2*G* and *SI Appendix*, Table S1, sheet 5). These proteins may be associated with mitochondria- or peroxisome-derived vesicles.

Our analyses indicate that the abundance of certain cargo proteins are more enriched in the vesicle fraction when the vesicle formation assay is performed in the presence of GTP than in the presence of GMPPNP. We found that 216 proteins showed more than twofold enrichment in the GTP group over the GMPPNP group (*P* < 0.05, Fig. 2*C*, area A and *SI Appendix*, Table S1, sheet 3). The *q* values of most of the identified hits (except one protein) are <0.05 (*SI Appendix*, Table S1, sheet 3). A total of 72% (156 in total) of proteins among the 216 identified proteins in area A are predicted by Uniprot to be transmembrane proteins: 110 of them are predicted to show Golgi localization, 11 of the predicted transmembrane proteins can be secreted, 21 proteins are predicted to show plasma membrane localization, and 8 show endosomal and lysosomal localization (Fig. 2*G* and *SI Appendix*, Table S1, sheet 6). Six of the identified proteins in area A are soluble secretory cargo proteins (Fig. 2*G*). We propose that these transmembrane proteins and soluble secretory proteins are cargo proteins that are packaged into vesicles in a GTP-hydrolysis–dependent manner.

### FAM84B/LRATD2 Associates with Vesicles in a GTP-Dependent Manner and Regulates ER-to-Golgi Transport of EGFR.

**Fig. 3.**
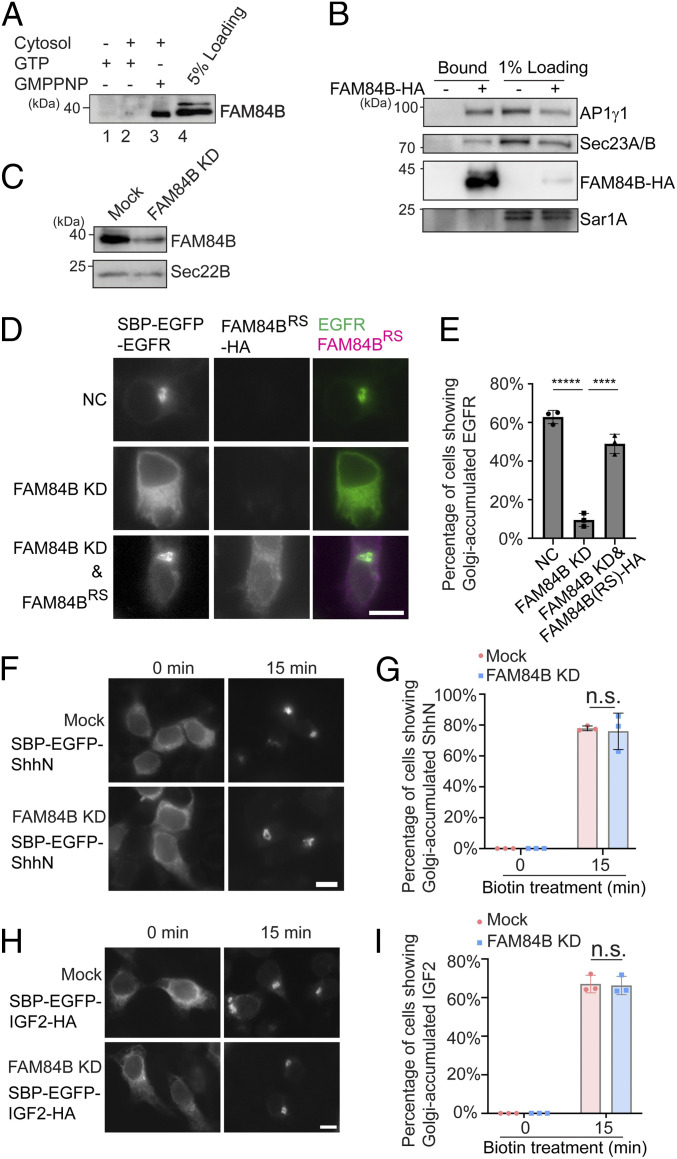
FAM84B/LRATD2 is recruited to vesicle membranes in a GTP-dependent manner, interacts with AP1γ1 and Sec23A/B, and regulates ER-to-Golgi transport of EGFR but not ShhN or IGF2. (*A*) The vesicle formation assay was performed using the indicated reagents. The proteins in the vesicle fraction were analyzed by Western blot. (*B*) HEK293T cells expressing the FAM84B-HA were treated in 2 mM dithiobis(succinimidyl propionate) (DSP), and the cell lysates were incubated with beads conjugated with HA antibodies. After incubation, the bound proteins were analyzed by Western blot using the indicated antibodies. (*C*) HEK293T cells were transfected with control siRNA or siRNA against FAM84B/LRATD2. Day 3 after transfection, cells were lysed and analyzed by Western blot. (*D*, *F*, and *H*) HEK293T cells were transfected with control siRNA or siRNA against FAM84B/LRATD2. Twenty-four hours after transfection, cells were transfected with plasmids encoding the indicted constructs. On day 3 after knockdown, cells were incubated with biotin and cycloheximide for 15 min and the localization of the cargo proteins was analyzed by fluorescent microscope. (Scale bar, 10 μm.) (*E*, *G*, and *I*) Quantifications of the percentage of cells showing Golgi-localized cargo proteins in each experimental group (mean ± SD; *n* = 3; >100 cells counted for each experiment). ****P < 0.0001; *****P < 0.00001; n.s., not significant. Data shown in *A–C* are representative examples of three biological repeats.

We next sought to perform experiments to verify one of the identified hits, FAM84B or LRAT domain-containing 2 (LRATD2), that associates with vesicle membranes in a GTP-dependent manner (Fig. 2*C*). FAM84B/LRATD2 is predicted to be found in clathrin-coated vesicles and partially colocalized with AP1γ1 (24, 25). Western blot analysis confirmed that FAM84B/LRATD2 was significantly enhanced in the vesicle fraction when the incubation was conducted in the presence of GMPPNP (Fig. 3*A*, compare lanes 2 and 3). HA-tagged FAM84B/LRATD2 (FAM84B-HA) was partially located at the cytoplasm and partially located at the juxtanuclear Golgi area colocalized with TGN46 (*SI Appendix*, Fig. S1*A*). After cells were treated with digitonin to remove the cytosolic pool, FAM84B/LRATD2 was partially located at the juxtanuclear Golgi area and partially located at the membrane structures in the cell periphery, presumably the ER (*SI Appendix*, Fig. S1 *B* and *C*). FAM84B-HA coimmunoprecipitated with AP1γ1 and Sec23A/B, but not Sar1A in the presence of a cross-linker (Fig. 3*B*).

We analyzed the role of FAM84B/LRATD2 in anterograde trafficking. We selected a transmembrane cargo protein, epidermal growth factor receptor (EGFR) and two soluble secretory cargo proteins, insulin-like growth factor II (IGF2) and the N-terminal fragment of sonic hedgehog (ShhN) (27). We analyzed trafficking of EGFR, ShhN, and IGF2 through a retention using selective hook (RUSH) transport assay (28, 29). In the RUSH assay, HEK293T cells were transfected with a plasmid encoding human EGFR or mouse ShhN or human IGF2 tagged with EGFP and the streptavidin binding peptide (SBP) (SBP-EGFP-EGFR or SBP-EGFP-ShhN or SBP-EGFP-IGF2-HA). This plasmid also encodes streptavidin fused to a C-terminal ER retention signal (Lys-Asp-Glu-Leu; Str-KDEL) (*SI Appendix*, Fig. S2 *A*, *H*, and *O*). Due to the binding between streptavidin and SBP, these cargo proteins were retained at the ER colocalized with an ER-located protein, the loop tail mutant of Vangl2 (HA-Vangl2^D255E^) (17) (*SI Appendix*, Fig. S2 *B–D* and *I–K*), or with an ER-located protein, Myc-atlastin-1 (*SI Appendix*, Fig. S2 *P–R*). In this condition, SBP-EGFP-ShhN was largely colocalized with the peripheral membranous structures marked by FAM84B-HA in the presence of digitonin (*SI Appendix*, Fig. S1*D*). When cells were incubated with biotin, SBP is released from streptavidin, thereby releasing the cargo proteins from the ER (*SI Appendix*, Fig. S2 *A*, *H*, and *O*). Thirty minutes after biotin treatment, SBP-EGFP-EGFR, SBP-EGFP-ShhN, and SBP-EGFP-IGF2-HA were delivered to the juxtanuclear area colocalized with the TGN marker, TGN46 (*SI Appendix*, Fig. S2 *E–G*, *L–N*, and *S–U*). Knockdown of FAM84B/LRATD2 caused a significant delay of EGFR transport from the ER to the Golgi in the RUSH transport system (Fig. 3*C* and *SI Appendix*, Fig. S3 *A* and *B*). The defects were rescued by expressing a siRNA-resistant construct of FAM84B-HA (Fig. 3 *D* and *E*). In contrast, knockdown of FAM84B/LRATD2 did not cause defects in the ER-to-Golgi transport of SBP-EGFP-ShhN and SBP-EGFP-IGF2-HA (Fig. 3 *F–I*). These analyses indicate that FAM84B/LRATD2 is important for ER-to-Golgi transport of EGFR but not ShhN and IGF2.

### Identification of Cargo Proteins and Cytosolic Proteins that Are Dependent on Sar1A for Their Association with Transport Vesicles.

**Fig. 4.**
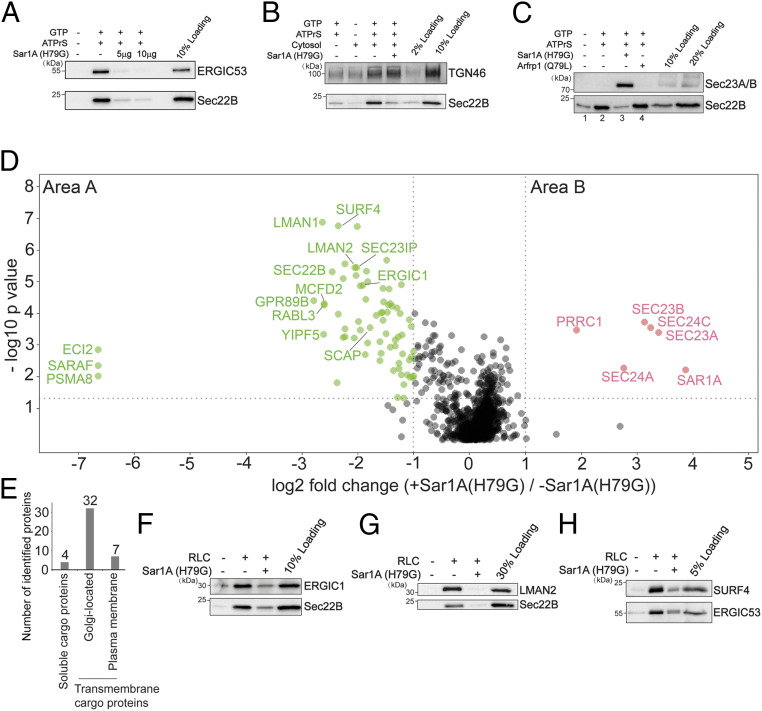
Identification of cargo proteins and cytosolic proteins that are dependent on Sar1A to be associated with transport vesicles. (*A–C*) The vesicle formation assay was performed using the indicated reagents. Vesicle fractions were analyzed by immunoblot. (*D*) The vesicle formation assay was performed in the presence or absence of Sar1A(H79G). The isolated vesicles in each experimental group were resuspended in RapiGest SF surfactant. The proteins in the vesicle fractions were trypsin digested and analyzed by label-free mass spectrometry. The log2 ratio of the abundance of each identified protein in the vesicles prepared in the presence of Sar1A(H79G) over that in the vesicles prepared in the absence of Sar1A(H79G) was plotted on the *x* axis and the −log10 *P* value of the difference was plotted on the *y* axis. (*E*) Number of proteins identified in area A in *D* categorized based on predictions from Uniprot. (*F–H*) The vesicle formation assay was performed using the indicated reagents. The vesicle fraction was analyzed by immunoblot. Data shown in *A–C* and *F–H* are representative examples of three biological repeats.

The experiment performed in the presence of GTP or GMPPNP revealed proteins that depend broadly on a group of GTP-binding proteins, such as Arf family proteins and Rab proteins, to be associated with vesicles. Next, we sought to utilize this assay to identify cytosolic proteins and cargo proteins that depend on a specific GTP-binding protein to be incorporated into transport vesicles. We focused our analysis on the Arf family member, Sar1, which initiates the assembly of the COPII coat at the ER (2). Sar1 has two isoforms in mammalian cells: Sar1A and Sar1B (2). The H79G mutation locks Sar1A in its GTP-bound form and inhibits COPII-dependent ER export (30). Consistent with previous reports, Sar1A(H79G) significantly abolished the vesicular capture of Sec22B and ERGIC53 (Fig. 4*A*). In contrast, Sar1A(H79G) did not interfere with the vesicular release of TGN46 (Fig. 4*B*), a cargo protein that cycles between the plasma membrane and the Golgi (31). Moreover, we found that Sar1A(H79G) enhanced the membrane association of Sec23A/B (Fig. 4*C*, compare lanes 3 and 2). In contrast, the dominant active form of another small GTPase, Arfrp1(Q79L), did not enhance the membrane association of Sec23A/B (Fig. 4*C*, compare lanes 4 and 3). These analyses suggest that our vesicle formation assay recapitulates the specific functions of Sar1A in mediating assembly of COPII coat proteins and in regulating packaging of cargo proteins into COPII vesicles.

We propose that proteins that are significantly reduced in the presence of Sar1A(H79G) are cargo proteins associated with COPII vesicles, and that cytosolic proteins that are significantly enhanced in the presence of Sar1A(H79G) are COPII coat proteins or proteins that directly or indirectly interact with COPII coat. We therefore performed our vesicle formation assay at a large scale in the presence or absence of Sar1A(H79G). Proteins in the vesicle fractions were quantified by label-free mass spectrometry (Fig. 4*D*). A total of 1,223 proteins were identified and quantified, all of which had two or more unique peptides (FDR < 0.01) and were successfully quantified in all of the three biological repeats (*SI Appendix*, Table S2, sheet 1). This analysis indicates that the vast majority of proteins that are significantly enriched in vesicles generated in the presence of Sar1A(H79G) are subunits of the COPII coat (more than twofold enrichment, *P* < 0.05, Fig. 4*D*, area B and *SI Appendix*, Table S2, sheet 2). However, a cytosolic protein in addition to COPII subunits was significantly enriched in the Sar1A(H79G) condition (Fig. 4*D*, area B and *SI Appendix*, Table S2, sheet 2, proteins identified using the protein sequence database of *H. sapiens* are indicated in round shapes and no additional proteins were identified using the database of *R. norvegicus*). All of the identified hits showed a *q* value of <0.05 (*SI Appendix*, Table S2, sheet 2).

Seventy-three proteins were identified with significant enrichment in the untreated group over the Sar1A(H79G) group (more than twofold enrichment, *P* < 0.05, Fig. 4*D*, area A and *SI Appendix*, Table S2, sheet 3). A total of 62 of these proteins showed a *q* value of <0.05 (*SI Appendix*, Table S2, sheet 3). A total of 50 of these 62 proteins are predicted to be transmembrane proteins and 4 were soluble cargo proteins that are secretory or located at the Golgi (Fig. 4*E* and *SI Appendix*, Table S2, sheet 4). Many of the transmembrane proteins were predicted to show plasma membrane and Golgi localization (Fig. 4*E* and *SI Appendix*, Table S2, sheet 4). None of these transmembrane proteins were predicted to show mitochondria or peroxisome localizations. All of the SNARE proteins identified in area B (Sec22A, Sec22B, STX5, GOSR2, and BET1) mediate ER-to-Golgi trafficking. A total of 33 among the 62 hits were identified to be associated with COPII vesicles reconstituted with purified COPII components containing specific Sec24 isoforms (13) (*SI Appendix*, Table S2, sheet 3). A total of 29 proteins were not identified in the previous study (*SI Appendix*, Table S2, sheet 3, highlighted with single asterisks) and many of them are transmembrane proteins that are predicted to show Golgi or plasma membrane localization. Western blot analysis confirmed that three transmembrane proteins, ERGIC1, SURF4, and LMAN2 were present in the vesicle fraction and their vesicular release was significantly reduced by Sar1A(H79G) (Fig. 4 *F–H*), indicating that they are packaged in COPII vesicles. Several cytosolic proteins were identified in area A, including RabL3, Sec23IP, and SCFD1. SCFD1 and Sec23IP have been shown to regulate ER-to-Golgi trafficking in mammalian cells (32–34). The role of RabL3 in ER-to-Golgi trafficking remains to be investigated.

In summary, these analyses revealed candidate cargo proteins that are packaged into COPII vesicles and candidate cytosolic proteins that are associated with COPII-coated vesicles. Moreover, these analyses indicate that our approach is robust in revealing effector proteins that are associated with GTP-bound Arf family proteins on vesicle membranes.

### PRRC1 Is Recruited to Vesicle Membranes by GTP-Bound Sar1A, Located at the ER Exit Sites and Regulates ER-to-Golgi Trafficking.

**Fig. 5.**
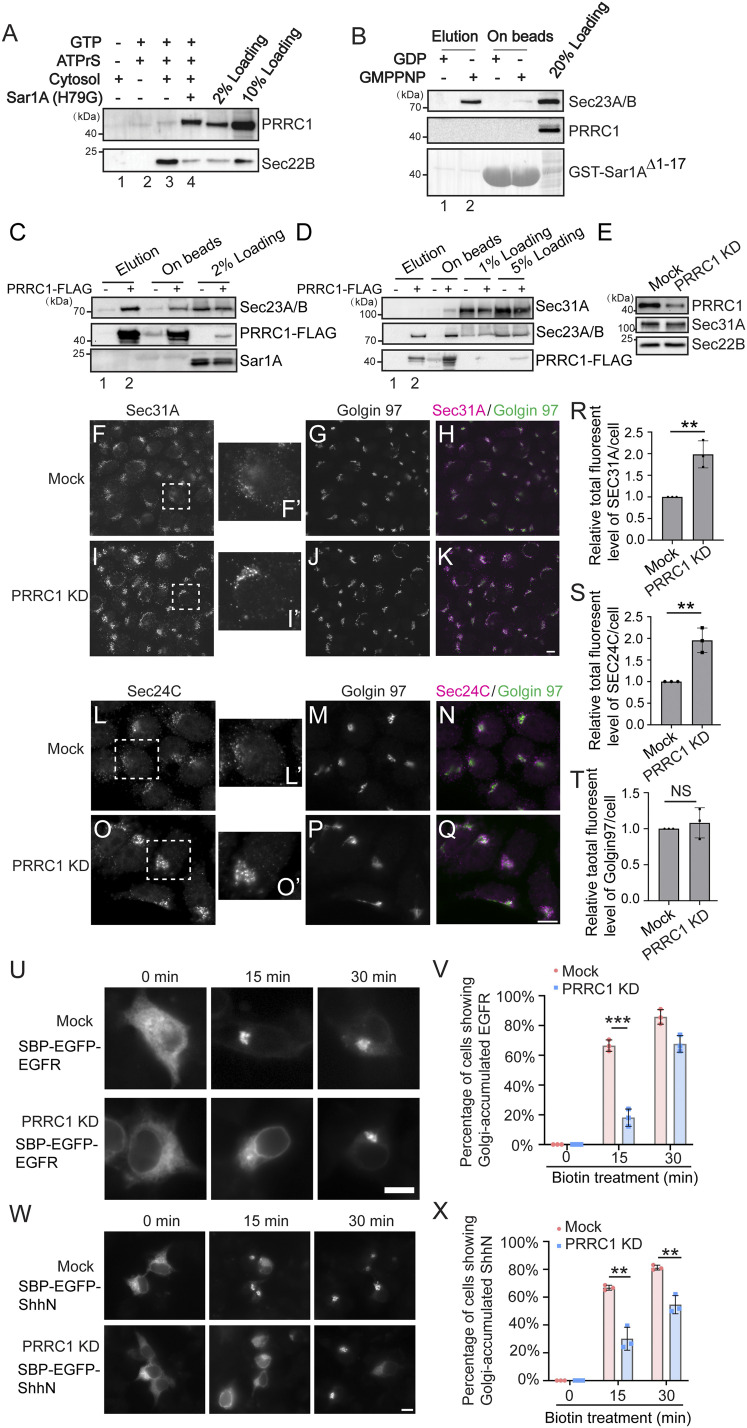
PRRC1 interacts with Sec23A/B and knockdown increases membrane association of Sec31A and Sec24C and decreases ER-to-Golgi transport of EGFR and ShhN. (*A*) The vesicle formation assay was performed using the indicated reagents. Vesicle fractions were analyzed by immunoblot. (*B*) GST-Sar1A^Δ1–17^ was loaded with GDP or GMPPNP and then incubated with rat liver cytosol. After incubation, proteins that bound to Sar1A in a nucleotide-dependent manner were eluted with EDTA. The eluted fraction and the proteins left on beads after elution were analyzed by immunoblot. (*C* and *D*) M2 agarose beads were incubated with cell lysates from HEK293T cells expressing the PRRC1-FLAG. After incubation, the bound proteins were eluted with 3× FLAG peptides and analyzed by Western blot using the indicated antibodies. (*E*) HEK293T cells were transfected with control siRNA or siRNA against PRRC1. Day 3 after transfection, cells were lysed and analyzed by Western blot. (*F*–*Q*) HEK293T cells were transfected with control siRNA (*F–H, L*–*N*) or siRNA against PRRC1 (*I–K, O*–*Q*). Day 3 after transfection, the localizations of Sec31A, Sec24C, and Golgin97 were analyzed by immunofluorescence. (Scale bar, 10 μm.) The magnified views of the indicated area in *F*, *I*, *L*, and *O* are shown in *F′*, *I′*, *L′*, and *O′*. (*R–T*) Quantifications of the total fluorescent level of Sec31A (*R*), Sec24C (*S*), and Golgin97 (*T*) per cell (mean ± SD; *n* = 3; >125 cells from nine random imaging fields counted for each experiment). In each experiment, the total fluorescent level was normalized to that in mock cells. **P < 0.01; NS, not significant. (*U* and *W*) HEK293T cells were transfected with control siRNA or siRNA against PRRC1. Twenty-four hours after transfection, cells were retransfected with plasmids encoding the indicated construct. On day 3 after knockdown, cells were incubated with biotin for the indicated time and the localization of the indicated protein was analyzed by fluorescent microscope. (Scale bar, 10 μm.) (*V* and *X*) Quantifications of the percentage of cells showing Golgi-localized EGFR or ShhN in cells treated with control siRNA or siRNA against PRRC1 (mean ± SD; *n* = 3; >100 cells counted for each experiment). ***P < 0.001; **P < 0.01. Data shown in *A*, *B*, *D*, and *E* are representative examples of three biological repeats.

A proline-rich domain-containing protein, PRRC1, was identified by our analysis to be recruited to vesicles by GTP-bound Sar1A (Fig. 4*D*, area B). The cellular function of PRRC1 is unknown. Western blot analysis confirmed that vesicles produced in the presence of Sar1A(H79G) contain higher levels of PRRC1 (Fig. 5*A*). We therefore tested whether PRRC1 was a binding partner of Sar1A utilizing a GST-pulldown approach. Purified GST-tagged human Sar1A with its N-terminal amphipathic helix removed (GST-Sar1A^Δ1–17^) was loaded with GDP or GMPPNP and then incubated with RLC. The concentration of RLC in the reaction mixture was equal to that used in the vesicle formation assay. After incubation, proteins that bound to Sar1A in a GTP-dependent manner were eluted with ethylenediaminetetraacetic acid (EDTA). Western blot analysis of the eluted fraction indicated that Sec23A/B was specifically detected in the eluate of GMPPNP-loaded but not GDP-loaded GST-Sar1A^Δ1–17^ immobilized on glutathione beads (Fig. 5*B*, compare lanes 1 and 2). In contrast, PRRC1 was not detected in the eluate of GMPPNP-loaded GST-Sar1A^Δ1–17^ (Fig. 5*B*). This GST pulldown occurred in the absence of a lipid bilayer, whereas the vesicle formation assay preserves the lipid bilayers of the ER and the Golgi. We propose that the vesicle formation assay has the advantage of revealing protein–protein interactions that take place on lipid bilayers. The interaction between PRRC1 and Sar1A might therefore require the intact membranes.

Coimmunoprecipitation experiments indicate that FLAG-tagged PRRC1 interacted with the inner COPII subunit, Sec23A/B, but not with the outer COPII subunit, Sec31A (Fig. 5 *C* and *D*, compare lanes 1 and 2). Knockdown of PRRC1 caused a significant enhancement of the total cellular immunofluorescence signal associated with Sec31A and Sec24C but not Golgin97 (Fig. 5 *E–Q*, quantifications in Fig. 5 *R–T*). Since the expression level of Sec31A in PRRC1 knockdown cells was similar to that in mock-treated cells (Fig. 5*E*), the enhanced fluorescence suggests that in the absence of PPRC1, enrichment of Sec31A and Sec24C at ER exit sites is enhanced. In addition, knockdown of PRRC1 caused a delay of transport of EGFR and ShhN from the ER to the Golgi in the RUSH transport system (Fig. 5 *U* and *W* and quantifications in Fig. 5 *V* and *X*), indicating that PRRC1 is important for ER-to-Golgi transport of both the transmembrane cargo protein, EGFR, and the soluble secretory cargo protein, ShhN.

We next analyzed the localization of a C-terminal HA-tagged PRRC1 (PRRC1-HA). When expressed at low levels, PRRC1-HA was partially located to the juxtanuclear area colocalized with the juxtanuclear-located Sec31A (*SI Appendix*, Fig. S4 *A–D* and magnified views in *SI Appendix*, Fig. S4 *A′*, *B′*, and *D′*). When expressed at high levels, PRRC1-HA was partially located at the nucleus and cytoplasm, and Sec31A and TGN46 were dispersed in the overexpressing cells (*SI Appendix*, Fig. S4 *E–H*). After cells were treated with digitonin in the presence of GMPPNP, the cytoplasmic pool of PRRC1-HA was dispersed and PRRC1 showed a clear juxtanuclear pattern that colocalized with the juxtanuclear-located Sec31A (*SI Appendix*, Fig. S4 *I–K*). Some of PRRC1-HA also located at the punctate structures at the cell periphery and the majority of these PRRC1 puncta colocalized with Sec31A (*SI Appendix*, Fig. S4 *I–K*, magnified views in *SI Appendix*, Fig. S4 *K′*–*K′′*), suggesting PRRC1 is located at ER exit sites (ERESs).

To test whether PRRC1 can be recruited to ERESs in a GTP-dependent manner, we performed a digitonin-permeabilized cell assay (35). HeLa cells were permeabilized, salt washed, and incubated with cytosol prepared from HEK293T cells expressing PRRC1-HA in the presence of GTP or GMPPNP. Both Sec31A and PRRC1-HA were efficiently recruited to the juxtanuclear area and to the punctate structures in the cell periphery in the presence of GMPPNP (*SI Appendix*, Fig. S4 *P–S*). In contrast, these proteins were not efficiently recruited to the semi-intact cells in the presence of GTP (*SI Appendix*, Fig. S4 *L–O* and quantifications in *SI Appendix*, Fig. S4 *W–X*). The majority of the peripheral punctate structures labeled by PRRC1-HA were colocalized with those labeled by Sec31A in the presence of GMPPNP (*SI Appendix*, Fig. S4 *P–S* and magnified views in *SI Appendix*, Fig. S4 *T′*–*V′′′′*). These results indicate that PRRC1 is recruited to ERESs in a GTP-dependent manner, and it modulates membrane association of the COPII coat and ER-to-Golgi trafficking.

### Identification of Potential Transmembrane Clients of COPI Vesicles.

We detected a significant enrichment of the COPI subunits in the vesicle fraction produced in the presence of GMPPNP (*SI Appendix*, Table S1, sheet 2), suggesting the vesicle fraction contains COPI vesicles. To identify the clients of COPI vesicles, we focused our analysis on transmembrane proteins in the vesicle fraction. We proposed that the membrane proteins that meet the following criteria are likely to be clients of COPI vesicles: 1) predicted by Uniprot to be localized at the Golgi but not located in endosomes, lysosomes, or plasma membrane; and 2) abundance in the vesicle fraction not significantly reduced by Sar1A(H79G). A total of 82 transmembrane proteins in the vesicle fraction fulfilled these criteria and are therefore likely to be clients of COPI vesicles (*SI Appendix*, Table S2, sheet 5). A total of 27 of these were previously identified in the COPI vesicle core proteome of HeLa cells (13) (*SI Appendix*, Table S2, sheet 5). Gene Ontology analysis of the 82 transmembrane proteins indicates that 45 proteins have transferase activities; 10 proteins have hydrolase activities; 5 proteins are SNAREs; and 6 proteins have transporter activities (*SI Appendix*, Table S2, sheet 5).

### Identification of Cargo Proteins that Depend on a Specific Cargo Receptor for Packaging into Transport Vesicles.

**Fig. 6.**
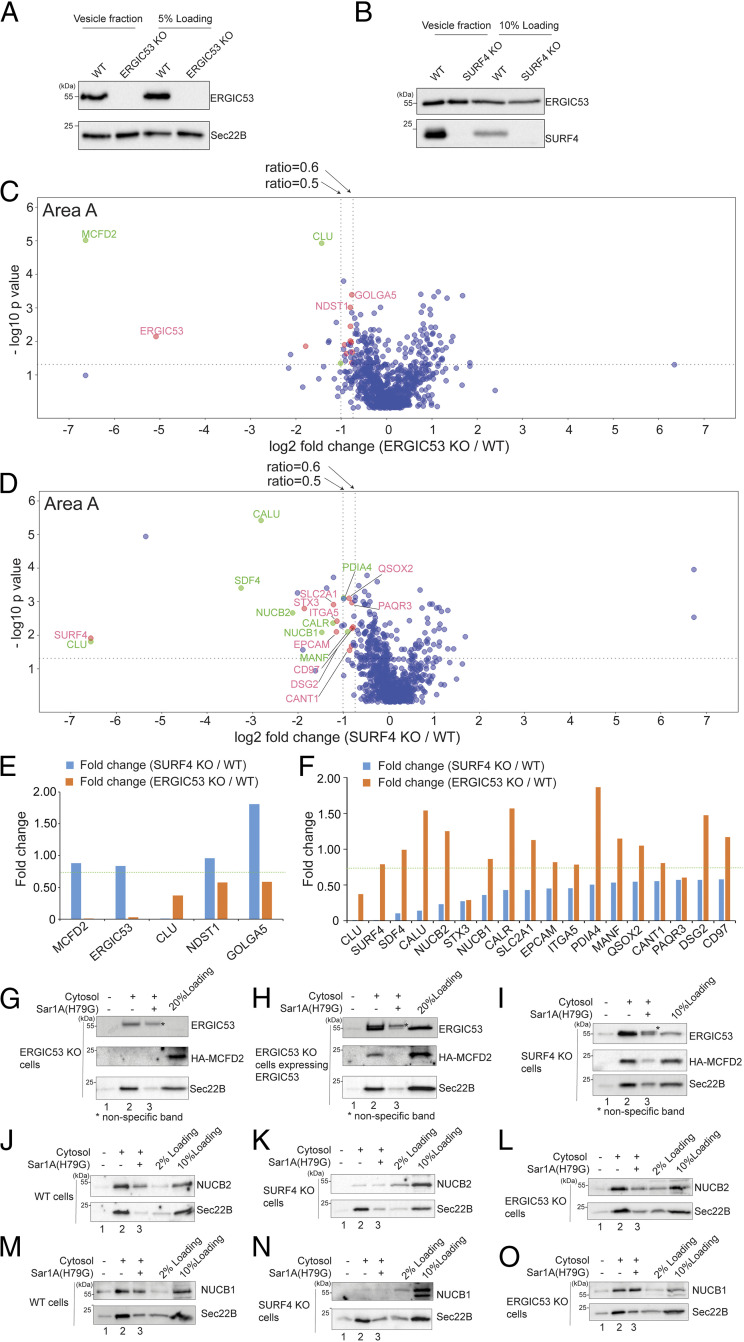
Identification of cargo proteins that depend on ERGIC53 or SURF4 for being packaged into transport vesicles. (*A* and *B*) The vesicle formation was performed using wild-type HEK293TRex cells and ERGIC53 KO HEK293TRex cells (*A*) or SURF4 KO HEK293TRex cells (*B*). The vesicle fraction was then analyzed by immunoblot. (*C* and *D*) The vesicle formation assay was performed using wild-type HEK293TRex cells and ERGIC53 KO HEK293TRex cells (*C*) or SURF4 KO HEK293TRex cells (*D*). The isolated vesicles in each experimental group were resuspended in RapiGest SF surfactant. The proteins in the vesicle fractions were trypsin digested and analyzed by label-free mass spectrometry. The log2 ratio of the abundance of each identified protein in the vesicles prepared from ERGIC53 KO or SURF4 KO cells over that in the vesicles prepared in wild-type cells was plotted on the *x* axis and the −log10 *P* value of the difference was plotted on the *y* axis. (*E*) The fold change of the identified ERGIC53 client was compared with the fold change of the abundance of these proteins in the vesicle fraction prepared from the WT cells and SURF4 KO cells. (*F*) The fold change of identified SURF4 client was compared with the fold change of the abundance of these proteins in the vesicle fraction prepared from the WT cells and ERGIC53 KO cells. Dotted line indicates the fold change of 0.75. (*G–O*) The vesicle formation was performed using the indicated cells. Vesicle fractions were then analyzed by immunoblot. Data shown in *G–O* are representative examples of three biological repeats. The single asterisks in panels *G*–*I* indicate the nonspecific bands recognized by anti-ERGIC53 antibodies.

Our vesicle formation assay uncovered several transmembrane cargo receptors that specifically depend on GTP hydrolysis by Sar1A for their enrichment into vesicles (Fig. 4*D*). Two of these proteins are ERGIC53 and SURF4 (Fig. 4 *A* and *H*). To reveal the client repertoire of ERGIC53 and SURF4, we performed the vesicle formation assay using donor membranes prepared from genome-engineered ERGIC53 knockout (KO) HEKTrex cells or SURF4 knockout HEKTrex cells. Western blot analysis confirmed the absence of ERGIC53 or SURF4 in the vesicles generated from the corresponding KO donor membranes (Fig. 6 *A* and *B*). We did not detect obvious changes in the localization of ERGIC53 in SURF4 KO cells (*SI Appendix*, Fig. S5 *A–C*) or changes in the localization of SURF4 in ERGIC53 KO cells (*SI Appendix*, Fig. S5 *D–F*). Similarly, KO cells showed no obvious defects in ER morphology, marked by HA-Vangl2^D255E^ (*SI Appendix, Fig. S5 G–I*) or the membrane association of COPII, marked by Sec31A (*SI Appendix, Fig. S5 J–L*). The *cis*-Golgi marker, GM130, was mainly located at the juxtanuclear region in wild-type (WT) and KO cells (*SI Appendix*, Fig. S5 *M–O*). GM130 in around 10% of WT cells and 30% of SURF4 KO cells or 30% of ERGIC53 KO cells located to some punctate structures in addition to the juxtanuclear localization (*SI Appendix*, highlighted by asterisks in *SI Appendix*, Fig. S5 *N* and *O*). Consistent with a previous report (36), we did not detect induction of the unfolded protein response (UPR) in SURF4 KO and ERGIC53 KO cells (*SI Appendix*, Fig. S5*P*).

Vesicles were generated from KO or WT donor membranes incubated with rat liver cytosol. Proteins in the vesicle fractions were then analyzed by label-free quantitative mass spectrometry. A total of 815 proteins were identified and quantified, all of which had two or more unique peptides (FDR < 0.01) and were successfully quantified in all three biological replicates of the experimental groups (*SI Appendix*, Table S3, sheet 1). In each case, several proteins were significantly reduced in the vesicle fraction of the KO reaction compared to WT (area A, Fig. 6 *C* and *D* and *SI Appendix*, Table S3, sheet 2). Transmembrane proteins that are predicted to show Golgi or plasma membrane localization are highlighted in red and soluble secretory proteins highlighted in green. Additional cargo proteins were significantly reduced in the vesicle fraction in the KO vesicles compared to WT when the threshold was changed from 0.5 to 0.6 (area A, Fig. 6 *C* and *D* and *SI Appendix*, Table S3, sheet 2).

Fourteen proteins, including ERGIC53, were underrepresented in the ERGIC53 KO condition relative to wild type (fold change < 0.6; *P* < 0.05, *SI Appendix*, Table S3, sheet 2). Five of them showed a *q* value of <0.05. Removing ERGIC53, we defined the remaining four proteins as ERGIC53 clients (highlighted in Fig. 6*C*, area A and *SI Appendix*, Table S3, sheet 2). One of these proteins, multiple coagulation factor deficiency protein 2 (MCFD2), is a known ERGIC53 interactor (37). The other three proteins may be novel interactors of ERGIC53. Among the remaining proteins with a *q* value of >0.05, coagulation factor V (FV) (*q* value = 0.1), is a known cargo client of ERGIC53 (*SI Appendix*, Table S3, sheet 2) (3). MCFD2 forms a complex with ERGIC53 to facilitate the transport of coagulation factors V and VIII (FVIII) from the ER to the Golgi (37, 38). We next examined the abundance of the four ERGIC53 clients in vesicles made from SURF4 KO cells (Fig. 6*E*). Three ERGIC53 clients were not similarly depleted in the SURF4 KO condition (Fig. 6*E*, above the green dotted line), suggesting that these cargo proteins are dependent on ERGIC53 but not SURF4 for efficient packaging into vesicles. Moreover, ERGIC53 is required for retention of MCFD2 in the early secretory transport pathway (39). Immunoblot analysis confirmed that packaging of HA-tagged MCFD2 into vesicles was abrogated in ERGIC53 KO cells (Fig. 6*G*, lane 2) but not in SURF4 KO cells (Fig. 6*I*, lane 2). Exogenously expressing ERGIC53 in ERGIC53 KO cells rescued packaging of MCFD2 into transport vesicles, and adding Sar1A(H79G) blocked this rescue (Fig. 6*H*, lanes 2 and 3).

Using similar criteria, 19 proteins were underrepresented in the SURF4 KO condition relative to wild type (fold change < 0.6; *P* < 0.05). Most of them (except one protein) showed a *q* value of <0.05 (*SI Appendix*, Table S3, sheet 2). Removing SURF4, we defined the remaining 17 proteins as SURF4 clients (highlighted in Fig. 6*D*, area A and *SI Appendix*, Table S3, sheet 2). A total of 16 out of 17 SURF4 clients were unaffected by the loss of ERGIC53 (Fig. 6*F*). Commercial antibodies against two of the top hits, NUCB1 and NUCB2, confirmed that these two proteins are packaged into vesicles, and the budding efficiency was reduced by Sar1A(H79G) (Fig. 6 *J* and *M*, compare lanes 2 and 3). Efficiency of NUCB1 and NUCB2 packaging was greatly reduced in SURF4 KO cells (Fig. 6 *K* and *N*, lane 2), whereas vesicular release of these two cargo proteins was not affected in ERGIC53 KO cells (Fig. 6 *L* and *O*, lane 2). Consistent with the observation from SURF4 KO cells, knockdown of SURF4 by siRNA (*SI Appendix*, Fig. S6*E*) also significantly reduced the efficiency of packaging of NUCB1 and NUCB2 into transport vesicles (*SI Appendix*, Fig. S6 *A* and *B*, compare lanes 2 and 6, quantifications in *SI Appendix*, Fig. S6 *C* and *D*). NUCB1 and NUCB2 coimmunoprecipitated with SURF4-HA in the presence of a cross-linker (*SI Appendix*, Fig. S6*F*), suggesting these cargo proteins interact with SURF4. Altogether, our analyses revealed specific transmembrane and soluble cargo proteins that depend on SURF4 or ERGIC53 to be packaged into transport vesicles. These analyses indicate that our method is a robust approach to reveal the clients of a specific transmembrane cargo receptor.

## Discussion

GTP-binding proteins, including Arf family proteins and Rab family proteins, play critical roles in mediating membrane recruitment of cytosolic factors to regulate cargo sorting and vesicle formation (14–16). Affinity chromatography is a traditional approach to identify these cytosolic factors. Here, we utilized the vesicle formation assay to investigate this aspect. A benefit of our approach is that membranes are preserved and our analysis indicates that this approach can reveal protein–protein interactions that take place on lipid bilayers. Through this approach, we identified cytosolic factors that are associated with vesicle membranes in a GTP-dependent manner or that interact with GTP-bound Sar1A on vesicle membranes. These cytosolic proteins may function as cargo adaptors or may associate with vesicle coats to regulate cargo sorting.

Two of these cytosolic proteins, FAM84B/LRATD2 and PRRC1, were shown to regulate ER-to-Golgi transport of newly synthesized EGFR. FAM84B/LRATD2 contains a LRAT (lecithin:retinal acyltransferase) domain. This domain is present in the H-Ras–like suppressor (HRASLS) family. The expression of FAM84B/LRATD2 is up-regulated during prostate cancer progression and in preclinical and esophageal squamous cell carcinoma tumors (40, 41). FAM84B/LRATD2 is shown to promote prostate tumorigenesis (42). PRRC1 is predicted to have protein kinase A regulatory subunit binding activity. Our results indicate that PRRC1 is recruited to ER exit sites in a GTP-dependent manner; associates with vesicle membranes in the presence of GTP-bound Sar1A; and down-regulates membrane recruitment of the COPII subunit, Sec24C and Sec31A. PRRC1 contains a proline-rich domain. The proline-rich region of Sec31 interacts with Sec23 (43–45), suggesting that PRRC1 may directly interact with Sec23 to perform its function. FAM84B/LRATD2 also contains a proline-rich domain but its function remains to be further investigated.

We found several further Arf and Rab proteins, besides Sar1A, whose abundances were significantly increased in the vesicle fraction produced in the presence of GMPPNP. It would be interesting to utilize our approach to reveal the cytosolic effector proteins that depend on these proteins to associate with transport vesicles. Our approach can also be performed in the presence of other vesicle-associated extrinsic factors, which would further reveal binding partners on vesicle membranes.

In addition to cytosolic proteins associated with vesicles, our approach is powerful in revealing cargo proteins that depend on a specific factor to be enriched into transport vesicles. In this study, we found cargo protein enrichment into vesicles depends on GTP hydrolysis by Sar1A. The protein composition of vesicles produced by purified COPII coats has been analyzed (13), which revealed several COPII clients that depend on specific isoforms of the COPII cargo-binding subunit, Sec24 (13). Release of the COPII clients, Sec22B and ERGIC53, into vesicles was greatly enhanced when the vesicle formation assay was performed using purified COPII supplemented with a low concentration of RLC (Fig. 1*K*). Using RLC as the source of cytosolic proteins, we found several non-ER resident cargo proteins that were not identified in the previous study. These cargo proteins may depend on cytosolic factors in addition to COPII in the RLC to be efficiently packaged into COPII vesicles.

Utilizing donor membranes from wild-type cells or cells depleted of a specific cargo receptor, we revealed clients of ERGIC53 and SURF4. Similar analysis will facilitate the identification of clients of other cargo receptors, thereby providing important information on the specificity of protein sorting and also revealing insight into the functional role of specific cellular factors. Another application of our approach is to analyze the protein composition of the vesicles produced from cells under different physiological conditions such as starvation. This could provide important insight into how vesicles contribute to establish and maintain a specific physiological condition. A caveat of our assay is that it relies on identification of cargo proteins that are actively produced by cells. Another caveat of our assay is that the donor membranes to produce vesicles are from digitonin-permeablized cells, so that the vesicles produced originate from multiple different organelles. It would be interesting to perform this assay in cell lines that highly secrete a variety of cellular factors to identify cargo proteins and utilize purified ER or Golgi membranes as the donor membranes for the vesicle formation assay.

In summary, our study demonstrates that the vesicle formation assay in combination with quantitative mass spectrometry analysis is powerful to identify cytosolic proteins that associate with vesicle membranes to regulate vesicular trafficking and to uncover cargo clients of a specific cellular factor, providing a robust tool to reveal insights into the secretory pathway.

## Materials and Methods

### Transfection, Immunofluorescence, and Permeabilized Cell Assays.

Transfection was performed using Lipofectamine 2000 (Invitrogen) or polyethyleneimine (PEI). Immunofluorescence was performed as described previously (20). Images were acquired with a Zeiss Axioobserver Z1 microscope system. Quantifications of the total fluorescence of Sec31A and Gogin97 were performed as described using ImageJ (46). Permeabilized cell assays were performed as described previously (35).

### In Vitro Vesicle Formation Assay.

In vitro vesicle formation assay was performed as described previously (20).

### Vesicle Immunogold Labeling and Negative Staining for Transmission Electron Microscopy Analysis.

Negative staining transmission electron microscopy (TEM) analysis and immunogold labeling were performed as described previously (47).

### Protein Purifications, Nucleotide Loading, and GST Pulldown.

Purification of His-tagged proteins from *Escherichia coli* was performed as described previously (48). Purification of GST-tagged protein was performed as described (16). The nucleotide loading and GST pulldown experiment was performed as described previously (14, 16).

### RUSH Assay.

HEK293T cells were transfected with plasmids encoding the RUSH construct of a specific cargo protein for 24 h. To release the RUSH construct of the cargo protein from the ER, cells were treated with 100 ng/μL cycloheximide (Sigma-Aldrich) and 40 μM D-biotin (Sigma-Aldrich) for the indicated time. Cells were then washed with phosphate-buffered saline (PBS), fixed with 4% paraformaldehyde (PFA), washed with PBS and mounted onto slides with ProLong Gold antifade mountant (Thermo Fisher).

### Sample Preparation for Label-Free Quantitative MS Analysis, Liquid Chromatography-MS Analysis, and MS Data Analysis.

These procedures were performed as described previously (19).

### Statistical Analysis of MS Data.

Student’s *t* test was used to compare the significant changes between two experimental groups based on the protein abundance values of the identified protein in three biological repeats in the two experimental groups. In addition, a permutation-based FDR with an s_0_ value of 0.01 was calculated using Perseus software (23). The identified proteins shown in Fig. 2*C*, areas A and B, Fig. 4*D*, areas A and B, Fig. 6*C*, area A, and Fig. 6*D*, area A were referred to as the identified hit proteins. The average abundance of the identified hit proteins that are less than 200,000 in both experimental groups was removed from the list of the identified hit proteins. The average abundance of each identified hit protein was calculated as the average of the absolute abundance of this protein in the three replicated experiments.

The abundance of proteins in the vesicle fraction isolated in the presence of GTP or GMPPNP was plotted using a histogram as previously reported (49). The enrichment analysis for categories of identified proteins was performed based on a Fisher’s exact test with a Benjamini–Hochberg FDR threshold of 0.02 using Perseus software.

### ATF6-Luciferase UPR Assay.

This assay was performed as described previously (51).

## Supplementary Material

Supplementary File

Supplementary File

Supplementary File

Supplementary File

## Data Availability

The mass spectrometry proteomics data have been deposited to the ProteomeXchange Consortium via the PRIDE (50) partner repository with the dataset identifier PXD026081.
