# Supplementary material for: An in vitro vesicle formation assay reveals cargo clients and factors that mediate vesicular trafficking
Source: Proc Natl Acad Sci U S A. 2021 Aug 25;118(35):e2101287118. doi: 10.1073/pnas.2101287118 (PMC8536394; doi:10.1073/pnas.2101287118)
Supplement: Supplementary File [file pnas.2101287118.sapp.pdf]

Supplemental Information (SI) Appendix

An *in vitro* vesicle formation assay reveals novel cargo clients and factors that mediate vesicular trafficking

Yan Huang [1,8], Haidi Yin [2,3,8], Baiying Li [5,8], Qian Wu [2,3], Yang Liu[1], Kristina Poljak[4], Julija Maldutyte[4], Xiao Tang[1], Mo Wang[1], Zhixiao Wu[1], Elizabeth A. Miller [4], Liwen Jiang [5], Zhong-Ping Yao [2,3,*], Yusong Guo [1,6,7,*]

1. Division of Life Science and State Key Laboratory of Molecular Neuroscience, The Hong Kong University of Science and Technology, Hong Kong, PR China
2. State Key Laboratory of Chemical Biology and Drug Discovery, Research Institute for Future Food and Department of Applied Biology and Chemical Technology, The Hong Kong Polytechnic University, Hung Hom, Kowloon, Hong Kong Special Administrative Region, China
3. State Key Laboratory of Chinese Medicine and Molecular Pharmacology (Incubation) and Shenzhen Key Laboratory of Food Biological Safety Control, Shenzhen Research Institute of Hong Kong Polytechnic University, Shenzhen 518057, China
4. Cell Biology Division, MRC Laboratory of Molecular Biology, CB2 0QH, Cambridge, UK
5. School of Life Sciences, Centre for Cell & Developmental Biology and State Key Laboratory of Agrobiotechnology, The Chinese University of Hong Kong, Shatin, New Territories, Hong Kong, China
6. Shenzhen Research Institute, Hong Kong University of Science and Technology, 518057, Shenzhen, China
7. Hong Kong Branch of Southern Marine Science and Engineering Guangdong Laboratory (Guangzhou), The Hong Kong University of Science and Technology, Clear Water Bay, Hong Kong, China
8. These authors contribute equally.

* Corresponding authors: guoyusong@ust.hk, zhongping.yao@polyu.edu.hk

Includes:

SI materials and methods

SI figures, Figure S1-S6

SI MATERIALS AND METHODS

Cell lines, antibodies and plasmids

HEK293T and HeLa cell lines were kindly provided by the University of California-Berkeley Cell Culture Facility. HEK 293Trex, HEK 293Trex ERGIC53 KO and HEK 293Trex SURF4 KO cell lines were contributed by Liz Miller's lab (MRC Laboratory of Molecular Biology, UK). All cell lines were tested negative for mycoplasma contamination. HeLa and HEK293T cells were maintained in Gibco Dulbecco's Modified Eagle Medium (DMEM) containing 10% fetal bovine serum (FBS) and 1% penicillin streptomycin mix. HEK 293Trex, HEK 293Trex ERGIC53 KO and HEK 293Trex SURF4 KO cell lines were maintained in Gibco DMEM containing 5 μg/mL blasticidin and 10% FBS. For temperature shift experiments, HEK293T, HEK 293Trex, HEK 293Trex ERGIC53 KO or HEK 293Trex SURF4 KO cells were incubated in Opti-MEM (Invitrogen, NY) containing 10% FBS at 15°C for 2.5 hrs to accumulate cargo proteins at the ER.

For CRISPR experiments, sgRNA sequences ligated into pX458 (pSpCas9 BB-2A-GFP) plasmids were purchased from GenScript. Transfections were performed with TransitIT-293 (Mirus Bio) per manufacturer's instructions. Clonal cell lines were derived by diluting cell suspensions to a single cell per well and expanding individual wells. Genotyping of clonal cell lines was performed by Sanger sequencing of target site PCR amplicons of genomic DNA isolated by Puregene kit (Quiagene). sgRNAs were as follows:

SURF4: 5'AGTCGCGCTGCTCGCTCCAC3' targeting exon 1;

ERGIC53: 5'TGACGGGGCTAGTCAAGCTT3' targeting exon 4.

Plasmids encoding His-tagged human Arfrp1 and Sar1A in pET28a vector and HA-tagged mouse Vangl2 in pCS2 vector were kindly provided by the Schekman lab (University of California, Berkeley). Plasmids encoding HA-tagged human FAM84B/LRATD2, human MCFD2 and human PRRC1 (transcript variant X1) in pcDNA3.1(+) vector were synthesized by Beijing Genomics Institution (BGI). Plasmids encoding human ERGIC53 were generated by inserting human ERGIC53 cDNA ORF from the cloning vector (Sino Biological Inc, number HG16166-U) into pCDNA3.1 vector. Plasmids encoding PRRC1-FLAG were generated by inserting human PRRC1 (transcript variant X1) into p3xFLAG-CMV-14 vector. Plasmids encoding Str-KDEL_SBP-EGFP-EGFR or Str-KDEL_SBP-EGFP-ShhN or Str-KDEL_SBP-EGFP-IGF2-HA were generated by replacing the DNA fragment encoding E-cadherin within the plasmid Str-KDEL_SBP-EGFP-Ecadherin (Addgene, Plasmid #65286) with a DNA fragment encoding human EGFR (amino acids 31-1210) or encoding mouse sonic hedgehog (amino acids 25-198) or encoding human IGF2 (amino acids 25-180 followed by 3xHA tag). Plasmids encoding Myc-tagged human atlastin-1 was kindly contributed by Dr. Junjie Hu's lab (IBP, China).

The polycolonal rabbit antibodies against Sec22B, ERGIC53, Sec23A/B, Sar1A for immunoblot analyses were gifts from Dr. Randy Schekman's lab (UC Berkeley). Rabbit anti-AP1μ1 antibodies were gifts from Dr. Jim Hurley's lab (UC Berkeley). Rabbit anti-SURF4 antibodies were gifts from Dr. Xiaowei Chen's lab (Peking University). The commercial antibodies were: Mouse anti-AP1γ1 (BD Bioscience, number 610385); Mouse anti-His (Qiagen, number 34660); Mouse anti-β-actin (Proteintech, number 60008-1-Ig); Rabbit anti-LMAN2 (Abcam, number ab124146); Rabbit anti-HA (Cell signaling technology, number 3724S); Sheep anti-TGN46 (AbD Serotec, number AHP500G); Rabbit anti-PRRC1 (Bethyl Laboratories, number A305-783A-T); Rabbit anti-FAM84B/LRATD2 (Proteintech, number 18421-1-AP); Rabbit anti-NUCB1 (Abcam, number ab206697); Rabbit anti-NUCB2 (Abcam, number ab229683); Rabbit anti-Sec31A (Bethyl, number A302-336A); Rabbit anti-AP1γ1 (Proteintech, number 13258-1-AP); Rabbit anti-Sec24C (Abcam, number ab122633), Rabbit anti-calreticulin (Abcam, number ab2907); Mouse anti-GM130 (BD Bioscience, number 610823), Rabbit anti-ERGIC53 for the immunofluorescence analysis (Sigma-Aldrich, number E1031).

Small interfering RNAs (siRNAs) were purchased from RUI BO (Guangzhou, China). Cells were harvested after 48 hrs or 72 hrs transfection.

The target sequence against PRRC1 is GACAAAACATTCAGTAGAA. The target sequence against FAM84B/LRATD2 is GCAACCAGGTGGAGAAATT. The target sequence against SURF4 is GCAGGAACTTCGTGCAGTA.

Transfection, immunofluorescence, permeabilized cell assays and immunoprecipitation

DNA constructs were transfected into HeLa, HEK293T, HEK 293Trex, HEK 293Trex ERGIC53 KO or HEK 293Trex SURF4 KO cells using lipofectamine 2000 (Invitrogen) or polyethyleneimine (PEI). Transfection of siRNA into HEK293T cells or HeLa cells was also performed using lipofectamine 2000 as described in the protocol provided by Invitrogen. The final working concentration of each siRNA is 40 nM.

Immunofluorescence was performed as described (1). Images were acquired with a Zeiss Axioobserver Z1 microscope system. Quantifications of the total fluorescence of Sec31A and Gogin97 were performed as described using Image J (2). For each experiment, a fixed threshold was manually selected that covers most of the signal on the original gray-scale images and applied to all images. Individual cells were then selected with the free-hand tool and the total above-threshold fluorescence was determined using the measure function. Permeabilized cell assays were performed as described (3).

Immunoprecipation of FAM84B-HA was performed using HEK 293T cells or FAM84B-HA overexpressed HEK293T cells. The cells were resuspended in PBS buffer and then treated with 2mM

DSP crosslinker (Thermo fisher Scientific, number:22586) for 30 min at RT. After treatment, unreacted DSP was quenched with 25 mM Tris (pH 7.4) for 15 min. Then the cells were resuspended in lysis buffer containing 50 mM Tris-HCl, 150mM NaCl, 2mM $CaCl_2$, 1%TX-100, protease inhibitors and 1mM DTT (pH 7.5) on ice for 30min. Anti-HA agarose beads (Thermo fisher Scientific, number:26181) were pre-blocked with blocking buffer (50mM Tris-HCl, 500mM NaCl, 2mM $CaCl_2$, 5% fat- free BSA, pH7.5) at 4 °C with rotation for 1 hr and then incubated with cell lysates for 4°C with rotation overnight. The beads were then washed with blocking buffer for 3 times and followed with lysis buffer for 3 times. Then the HA agarose beads were boiled in reduced sample buffer and analyzed by immunoblot.

Immunoprecipation of PRRC1-FLAG was performed using HEK293T cells or PRRC1-FLAG overexpressed HEK293T cells. The cells were lysed with KOAc buffer containing 0.5%Triton X-100, protease inhibitors and 1mM DTT for 30 min on ice. After 10min 14000 g centrifugation, the cell lysate supernatant was incubated with anti-FLAG M2 agarose beads (Sigma-Aldrich, number A2220) at 4 °C with rotation overnight. The beads were then washed with KOAc buffer and eluted with KOAc buffer containing 0.6 mg/mL 3x FLAG peptides. The eluted fraction and on beads fraction were analyzed by immunoblot.

***In vitro* vesicle formation assay**

In vitro vesicle formation assay was performed as described (1). HEK293T, HEK 293Trex, HEK 293Trex ERGIC53 KO or HEK 293Trex SURF4 KO cells were permeabilized in ice cold KOAc buffer (110 mM potassium acetate, 2.5 mM magnesium acetate, 20 mM Hepes, pH 7.2) containing 40 mg/mL digitonin on ice for 5 min. The permeabilized cells were collected by centrifugation at 300 g for 3 min at 4 °C, washed with KOAc buffer and resuspended in KOAc buffer. The semi-intact cells were then incubated at 30 °C with 2 mg/mL RLC, 200 μM GTP or GMPPNP (Wako, number SAH3766) and an ATP regeneration system consisting of 40 mM creatine phosphate (Roche, number 10621722001), 0.2mg/mL of creatine phosphokinase (Roche, number 10736988001), and 1mM ATP (Sigma, number A2383). After a 1 hr incubation, the reaction mixture was centrifuged at 14000 g at 4 °C for 20 min to remove the ER and Golgi membranes, nucleus as well as other cell debris from the reaction mixture. The supernatant fraction containing the released vesicles was resuspended in 35% Opti-Prep and overlaid with 30% Opti-Prep. 50 μL KOAc buffer was added on the top of the step gradient of Opti-Prep. The Opti-Prep gradients were then centrifuged at 100000 g in a TLS55 (Beckman) or S55S (Hitachi) rotor at 4°C for 1.5 hr. After centrifugation, the top fraction (the vesicle fraction) was collected and analyzed by negative stain electron microscope, Coomassie staining, western blot or mass spectrometry.

Vesicle immuno-gold labeling and negative staining for transmission electron microscopy analysis

Negative staining transmission electron microscopy (TEM) analysis was performed essentially as described previously (4). Briefly, 10 µl of the vesicle fraction was applied onto the pre-glow discharged carbon-coated 400-mesh copper grids (EMR, 22-1MC040-50) for 10 min before further labelling or negative staining. The glow discharge was performed using the PELCO easiGlow^{TM} Glow Discharge Cleaning System (Ted Pella, Inc., Redding, CA). For negative staining, extra liquid was removed by filter paper after 10 min vesicle sedimentation on the grid, followed by negatively stained by 10 µl 2% uranyl acetate (UA) for 2 min. Extra UA liquid was removed and the grids were further air dried before TEM observation. For immuno-gold labelling, vesicles on grid were first blocked by 0.1-1% BSA in PBS, followed by primary antibody incubation at optimized concentrations for 2-3 hr. The grids were briefly washed by 0.1% BSA before applying goat anti-rabbit 6 nm gold-coupled secondary antibodies (EMS, 25104) at 1:20 dilution for 1 hr. Immunogold-labelled vesicles were then negatively stained as previously described before TEM observation. The quantification and comparison of vesicle size or number were performed in a same area size for images from different groups for each experiment as indicated.

Protein purifications, nucleotide loading and GST pull down

To purify His-tagged proteins from E. coli, cells expressing His-tagged constructs were grown to the optical density at 600 nm (OD600) at 0.6-0.8 in lysogeny broth (LB) and induced with 0.5mM isopropyl β-D-1-thiogalactopyranoside (IPTG) (Sigma, number i5502) at 25 °C for 4 hr for the protein expression. The cells were lysed with lysis buffer containing 10mM imidazole and 1mg/mL lysozyme in 2x PBS buffer (274mM NaCl, 5.4mM KCl, 20mM Na_2HPO_4, 3.6mM KH_2PO_4, pH7.4), followed by addition of TX-100 to 0.5% and sonication. The lysate was centrifuged at 100 kg for 10 min to remove cell debris and then the supernatant was incubated with HisPur™ Ni-NTA Resin overnight. The protein was eluted by elution buffer containing 250 mM imidazole, protease inhibitors and 1mM DTT in KOAc buffer. The eluted protein was dialyzed against KOAc buffer.

Purification of GST-tagged protein was performed as described (5). The nucleotide loading and GST pull down experiment was performed as described (5, 6). Briefly, 10 µL glutathione beads bearing around 10 µg GST-Sar1A$^{\Delta 1\text{-}17}$ were washed 3 times with 200 µL Nucleotide Exchange (NE) buffer (20 mM HEPES, pH 7.4, 100 mM KCl, 2mM EDTA, 1mM $MgCl_2$, 1 mM DTT) then the beads were incubated with 150 µl NE buffer containing 500 µM GDP or GMPPNP at 37 °C for 90 min. After incubation, the beads were washed 3 times with 200 µL Nucleotide Stabilization (NS) buffer (20 mM HEPES, pH 7.4, 100 mM KCl, 5mM $MgCl_2$, 1 mM DTT) then incubated with 150 µL NS

buffer containing 500 μM GMPPNP or GDP at 37 °C for 45 min. After incubation, the beads were incubated with 2 mg/mL RLC containing 100 μM GDP or GMPPNP at 4 °C for 2hrs. The beads were then washed six times with 500 μL NS buffer and one time with 500 μL NS buffer without magnesium and then the bound proteins were eluted with elution buffer (20mM Hepes, 1.5M KCl, 0.5% Triton-100, 20mM EDTA) and analyzed by immunoblot. All of the incubations were performed in the presence of proteinase inhibitors.

Sample preparation for label-free quantitative MS analysis

0.1% RapiGest SF Surfactant was dissolved in 50 mM triethylammonium bicarbonate (TEAB) (Sigma-Aldrich, number T7408). Vesicle samples were resuspended in 0.1% RapiGest by vortexing. The same volume of 8M urea dissolved in 50 mM TEAB buffer was added into the vesicle sample. The sample was then reduced with 10mM TCEP at 37°C for 1 hr and alkylated with 20mM iodoacetamide (IAA) at room temperature in the dark for 30 min, followed by the dilution with 50 mM TEAB for 4 times and the digestion with sequencing grade modified trypsin (Promega, number V511A) at 37 °C for 20 hr. To stop the trypsin digestion and to remove the surfactant, the pH of vesicle samples was adjusted to 2.5-3.0 by adding 10% trifluoroacetic acid (TFA). The degraded surfactant was removed by centrifugation. Samples were dried by speed vacuum. Next, samples were desalted with pierce C18 spin column (Thermo fisher Scientific, number 89870). Subsequently, the samples were dried again to be analyzed by the mass spectrometry.

Liquid chromatography-MS analysis

Mass spectrometry and data analysis was performed as described (7). Briefly, LC separation was performed using an Acclaim PepMap RSLC C18 capillary column (75 μm × 25 cm; 2 μm particles, 100 Å) (Thermo fisher Scientific, San Jose, CA). Gradient elution was performed using an Ultimate 3000 nanoLC system (Thermo fisher Scientific, San Jose, CA). The flow rate was set at 300 nl/min. Mobile phase A was 0.1% formic acid in water and mobile phase B was 0.1% formic acid in acetonitrile. The analytical gradient lasted for 90 min including the following steps: 1) 10 min of equilibration with 3% B; 2) the composition of solvent B was increased from 3% to 7% in 2 min, from 7% to 20% in 50 min, and from 20% to 30% in 2 min; 3) a washing and equilibration step when solvent B was increased to 80% in 1 min and was held for 8 min; 4) the composition of solvent B was returned to 3% in 0.1 min and was held for 17 min.

Analysis was performed using an Orbitrap Fusion Lumos mass spectrometer (Thermo fisher Scientific, San Jose, CA) operating in positive ion mode. The ESI spray voltage was set at 2300 KV and the ion transfer tube temperature was set at 300°C. MS and MS/MS scans were performed using

high resolution Orbitrap, with resolution at 60K and 15K, respectively. Data-dependent acquisition (DDA) mode was performed with a cycle time of 3 s. The mass range of the full MS scan defines m/z 400 to 1600, and in MS/MS starts from m/z 110. The collision energy was set at 30%. Three biological repeats of each sample were performed.

MS data analysis

Proteome Discoverer 2.2.0.388 was used for protein identification and quantification with the following settings: (1) fixed modification: cysteine carbamidomethylation (+57.021 Da); (2) dynamic modification: methionine oxidation (+15.995 Da) and acetylation (+42.011) at the N terminus of the protein; (3) trypsin was used for digestion with one missed cleavage allowed; (4) peptide ion tolerance: 10 ppm; (5) fragment ion tolerance: 20 ppm; (6) the protein sequence database of *Homo sapiens* was downloaded from Uniprot (updated 11-2018) for database searching and identification with a false discovery rate (FDR) <0.01; (7) the minora algorithm based label-free quantification (LFQ) was performed using the intensity of precursor ions; (8) unique and razor peptides of proteins were selected to calculate the abundance ratio of proteins; (9)The abundance ratio of each identified proteins was determined using pairwise-ratio based calculation where the median value of the abundance ratio of all matched peptides from three biological repeats was used as the abundance ratio of the identified protein and the maximum allowed fold change was set as 100; (10) The protein abundance was determined by the average intensities of top three unique peptides (if <3 peptides can be quantified, the average intensity of these peptides was used).

Proteins with two or more unique peptides and successfully quantified in all of the three biological repeats of at least one experimental group were selected for quantitative analysis. The protein abundance was normalized to the median value of each sample to correct experimental bias based on the total protein amount before further statistical comparison.

ATF6-Luciferase Unfolded Protein Response (UPR) assay (8)

HEK293 Flp-In Trex WT, SURF4 KO and LMAN1 KO cells were seeded at 5000 cells/well in a 96-well plate (Corning, 3610). The following day cells were transfected in replicates of 6 with a plasmid containing 5 tandem ATF6 promoter elements upstream of Renilla Firefly luciferase ORF (8). The next day control wells were treated with the following UPR inducers: tunicamycin (2.5 ug/mL), thapsigargin (0.2 uM) and increasing concentrations of dithiothreitol (DTT) (0.2-1 mM). After 16h of antibiotic treatment media in all wells was aspirated, exchanged for complete DMEM containing 100 uM Luciferin (Carbosynth, L-8220), incubated at 37C for 30 min and assayed using a luminescence plate reader.

SI REFERENCES

1. T. Ma et al., A mechanism for differential sorting of the planar cell polarity proteins Frizzled6 and Vangl2 at the trans-Golgi network. J Biol Chem 293, 8410-8427 (2018).
2. Y. Guo, A. D. Linstedt, COPII-Golgi protein interactions regulate COPII coat assembly and Golgi size. J Cell Biol 174, 53-63 (2006).
3. F. Yang, T. Li, Z. Peng, Y. Liu, Y. Guo, The amphipathic helices of Arfrp1 and Arl14 are sufficient to determine subcellular localizations. J Biol Chem 10.1074/jbc.RA120.014999 (2020).
4. Y. C. Tse et al., Identification of multivesicular bodies as prevacuolar compartments in Nicotiana tabacum BY-2 cells. Plant Cell 16, 672-693 (2004).
5. Y. Guo, G. Zanetti, R. Schekman, A novel GTP-binding protein-adaptor protein complex responsible for export of Vangl2 from the trans Golgi network. Elife 2, e00160 (2013).
6. S. Christoforidis, M. Zerial, Purification and identification of novel Rab effectors using affinity chromatography. Methods 20, 403-410 (2000).
7. L. Niu et al., Atlastin-mediated membrane tethering is critical for cargo mobility and exit from the endoplasmic reticulum. Proc Natl Acad Sci U S A 116, 14029-14038 (2019).
8. J. Shen, R. Prywes, ER stress signaling by regulated proteolysis of ATF6. Methods 35, 382-389 (2005).

Figure S1

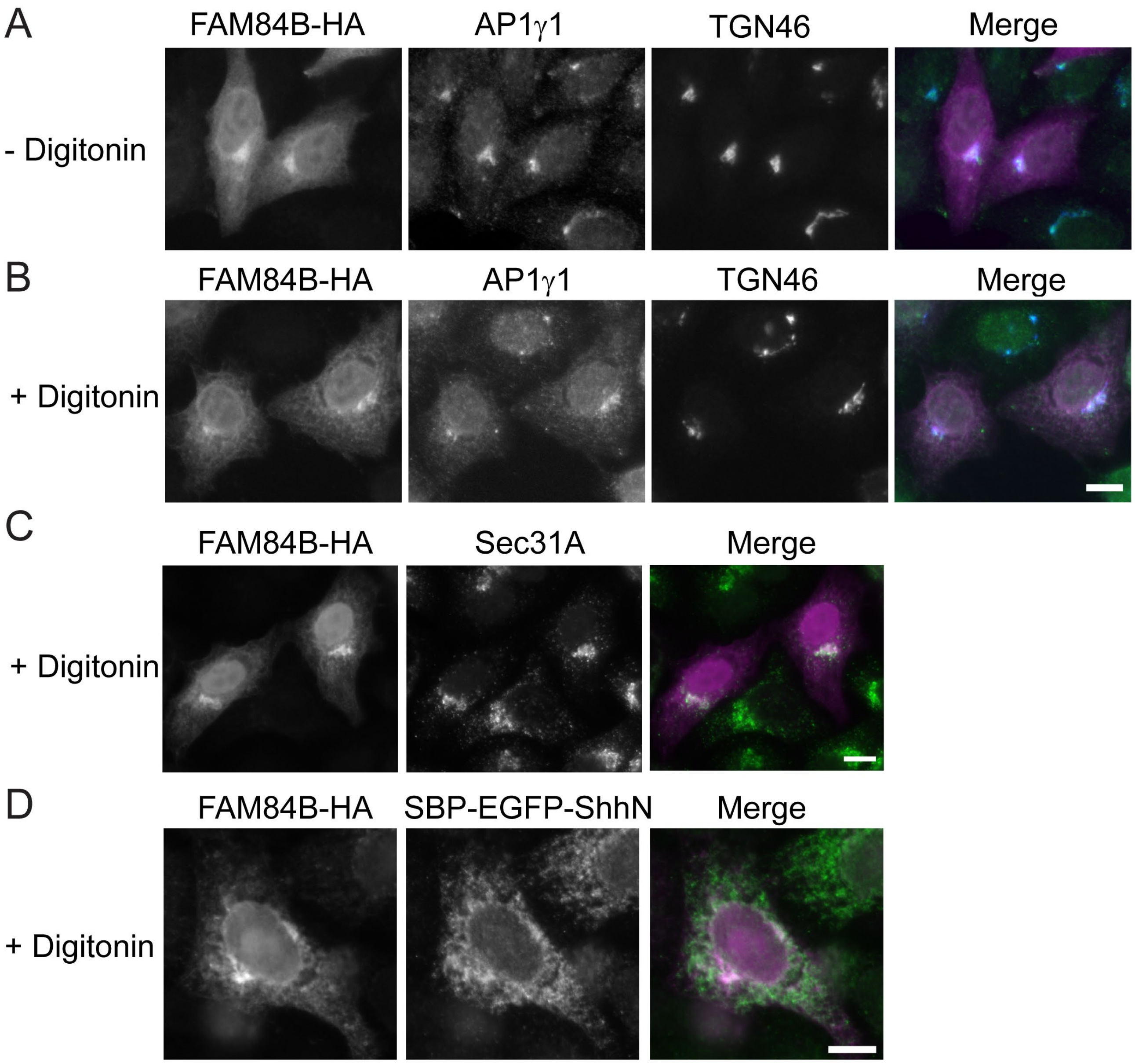

Figure S1. Localization of FAM84B-HA.

HeLa cells were transfected with FAM84B-HA. Day 1 after transfection, cells were untreated (A) or treated with digitonin (B-D) and the localizations of the indicated proteins were analyzed by immunofluorescence. *Scale bar*, 10 μm.

Figure S2

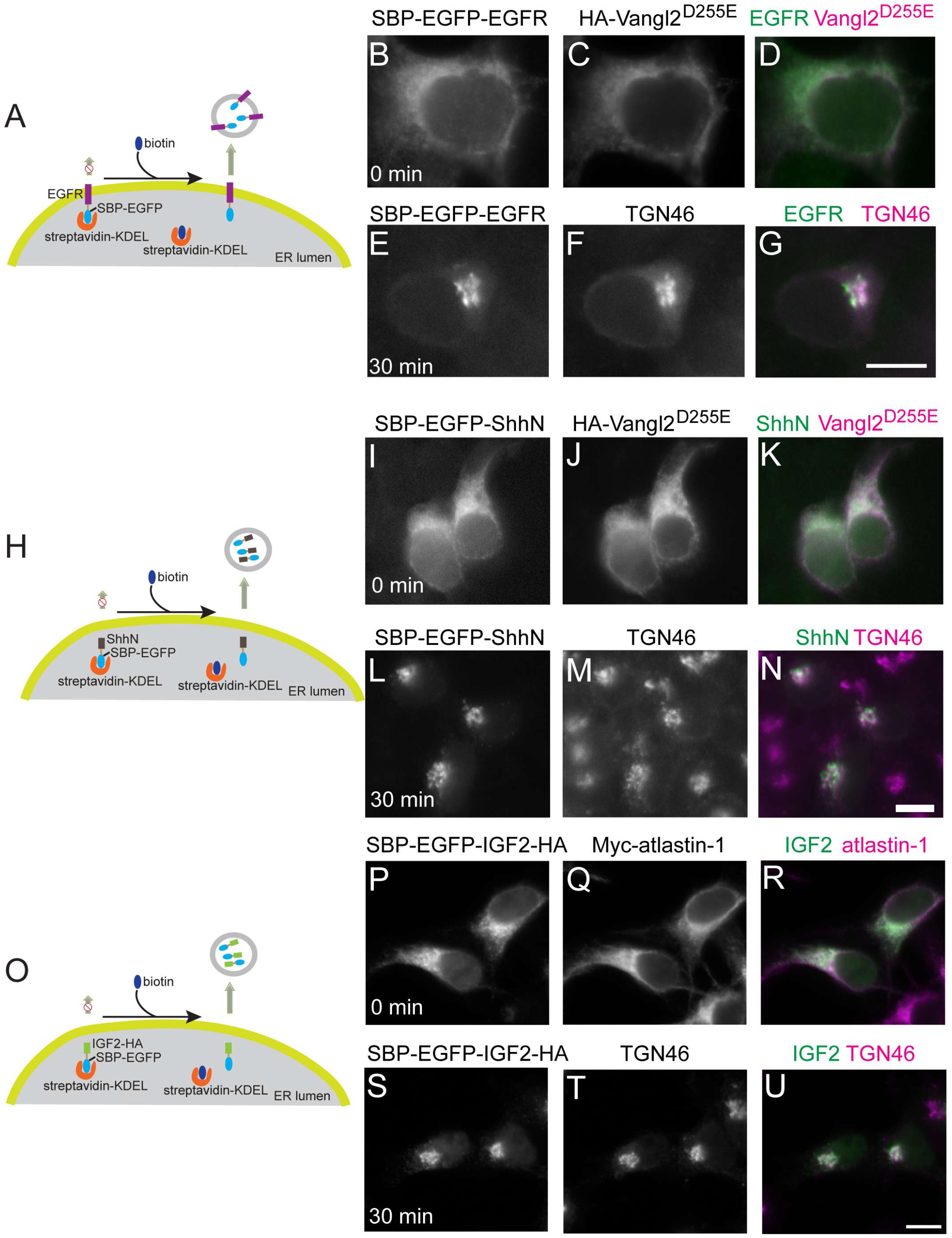

Figure S2. The RUSH transport assay. A, H and O. Diagram demonstrating the RUSH assay to monitor the trafficking of SBP-EGFP-EGFR, SBP-EGFP-ShhN and SBP-EGFP-IGF2-HA. **B-G, I-N and P-U.** HEK293T cells were transfected with plasmids encoding the indicated constructs. On day 3 after knockdown, cells were incubated with biotin and cycloheximide for the indicated time and the localization of the indicated constructs was analyzed by immunofluorescence. Scale bar, 10 µm.

Figure S3

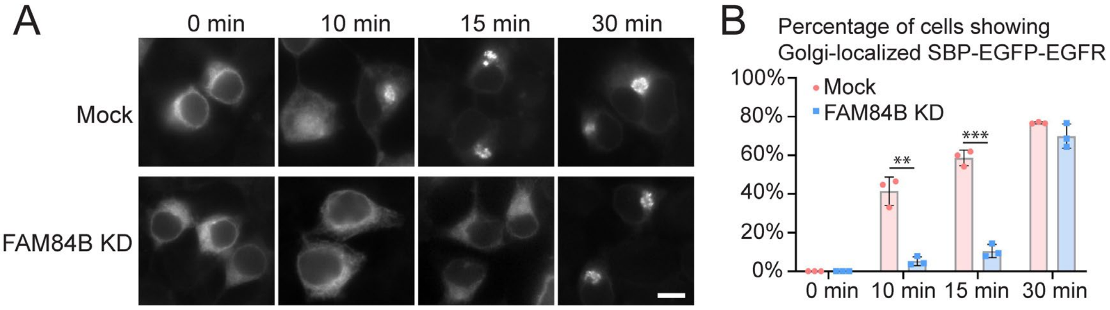

Figure S3. FAM84B/LRATD2 regulates ER-to-Golgi trafficking of EGFR. A. HEK293T cells were transfected with control siRNA or siRNA against FAM84B/LRATD2. 24hr after transfection, cells were re-transfected with plasmids encoding SBP-EGFP-EGFR. On day 3 after knockdown, cells were incubated with biotin and cycloheximide for the indicated time and the localization of EGFR was analyzed by fluorescent microscope. Scale bar, 10 µm. **B.** Quantification of the percentage of cells showing Golgi-localized EGFR in cells treated with control siRNA or siRNA against FAM84B/LRATD2 (mean ± S.D.; n = 3; >100 cells counted for each experiment). **, p<0.01; ***, p < 0.001.

Figure S4

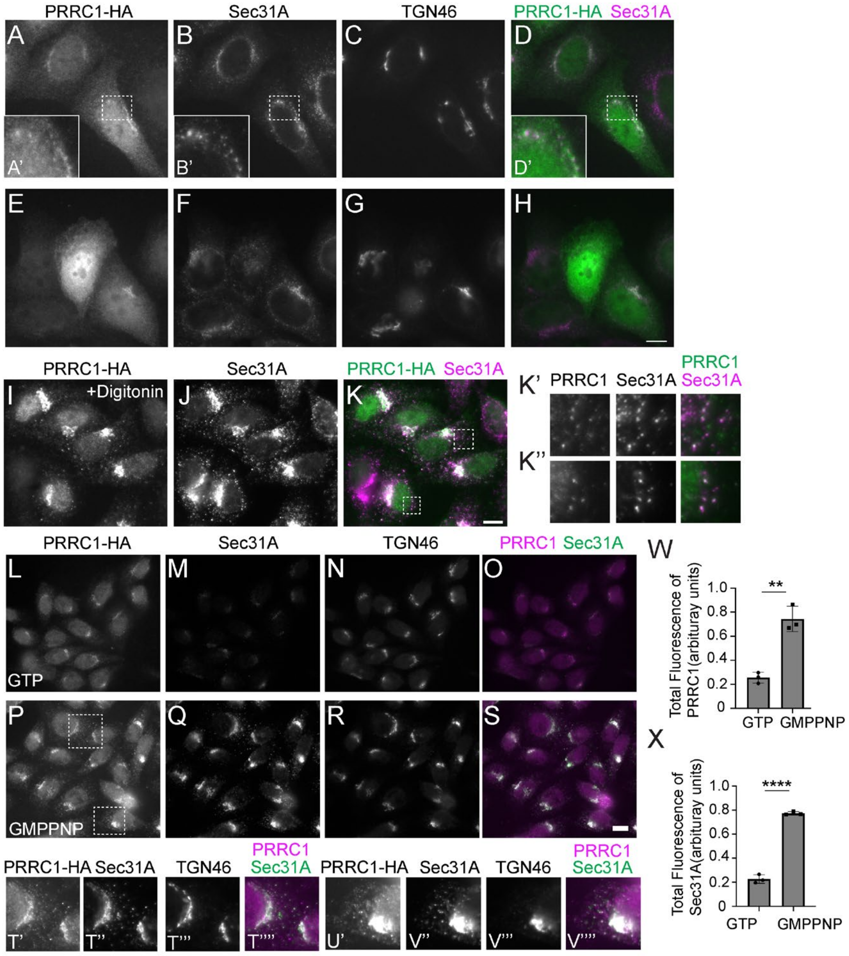

Figure S4. PRRC1 is recruited to the ER exit sites in a GTP-dependent manner.

A-K''. HeLa cells were transfected with PRRC1-HA. Day 1 after transfection, cells were untreated (A-H) or treated with digitonin in the presence of GMPPNP (I-K''), and then the localizations of the indicated proteins were analyzed by immunofluorescence. *Scale bar*, 10 µm. The magnified views of the indicated areas in panels A, B, D and K were shown in panels A', B', D', K' and K'' and the brightness is adjusted to highlight the punctate structures in these magnified views. **L-V''''.** HeLa cells were permeabilized by digitonin and incubated with the cytosol prepared from HEK293T cells expressing PRRC1-HA in the presence of GTP (L-O) or GMPPNP (P-V''''). After incubation, the localization of the indicated proteins were analyzed by immunofluorescence. Scale bar, 10 µm. The magnified views of the indicated areas in panel P are shown in panels T'-V'''' and the brightness is adjusted to highlight the punctate structures in these magnified views. **W-X.** The total fluorescence of the indicated proteins was quantified in the experiment performed in the presence of the indicated nucleotides (n=3, mean ±S.D., >190 cells from 6 random imaging fields counted for each experiment). **, $p<0.01$; ****, $p<0.0001$.

Figure S5

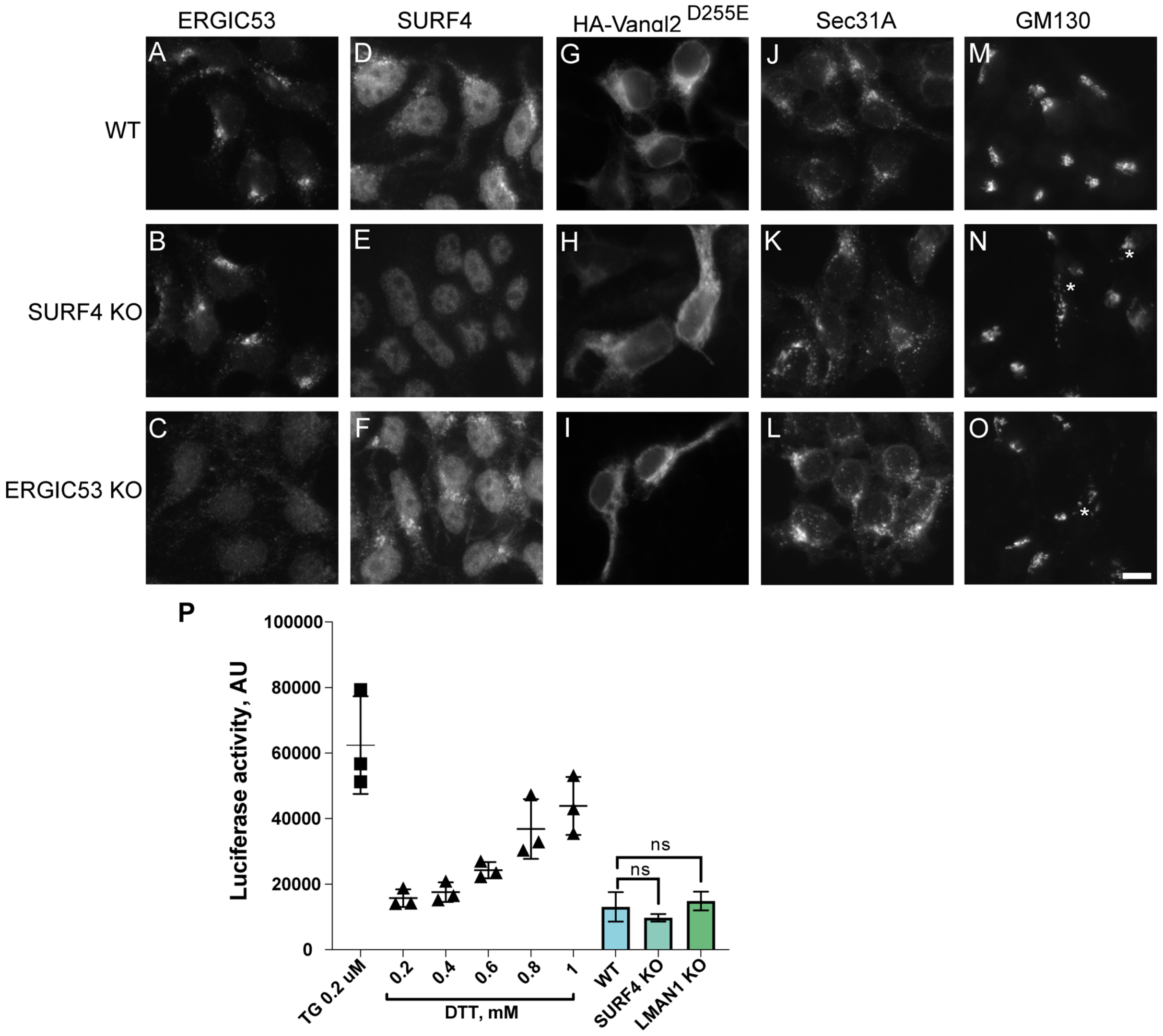

Figure S5. Analyses of the Unfolded Protein Response (UPR) and localizations of ERGIC53, SURF4, HA-Vangl2 ^{D255E}, Sec31A and GM130 in wt, SURF4 KO and ERGIC53 KO cells. A-O. The localizations of the indicated proteins in wt, SURF4 KO and ERGIC53 KO HEK293Trex cells were analyzed by immunofluorescence. Scale bar, 10 µm. Asterisks in panels N and O highlight cells showing punctate patterns of GM130. **P.** ATF6-Luciferase UPR assay was performed using the indicated cells in the presence or absence of the indicated UPR inducers.

Figure S6

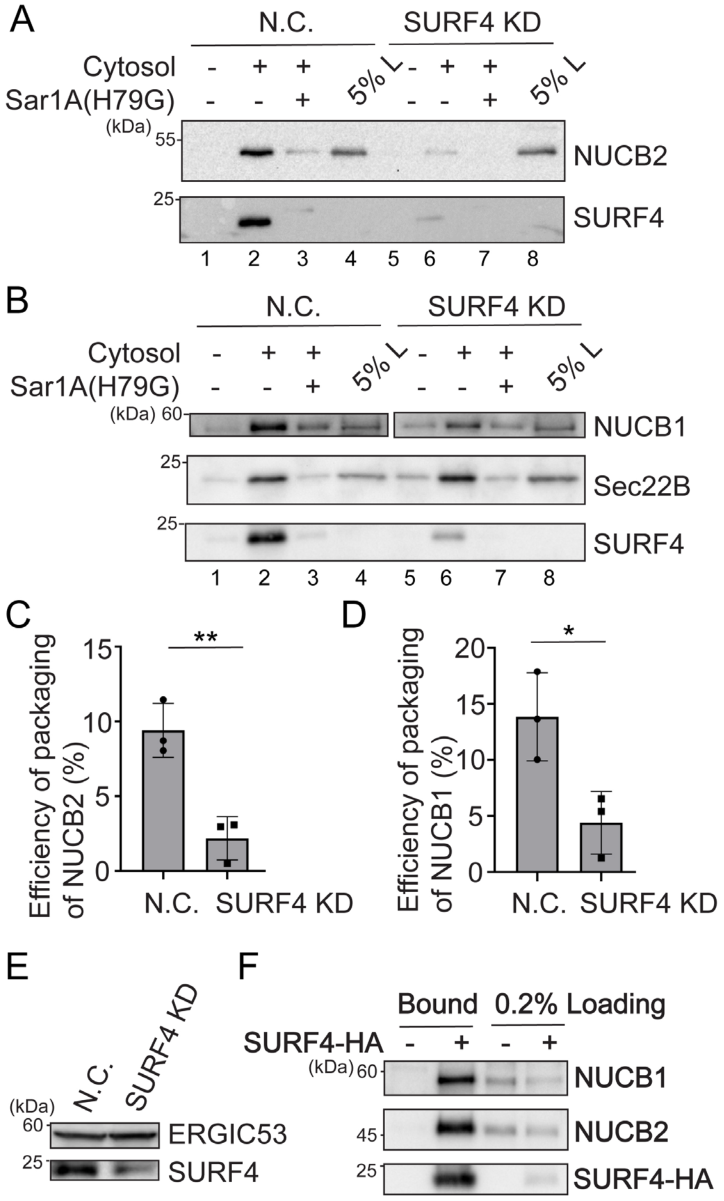

Figure S6. SURF4 interacts with NUCB1 and NUCB2, and regulates the packaging of NUCB1 and NUCB2 into transport vesicles. A-B. HEK293T were transfected with negative control (N.C.) siRNA or siRNA against SURF4 (SURF4 KD). On day 3 after transfection, the vesicle formation was performed using the indicated cells and reagents. Vesicle fractions were then analyzed by immunoblot. **C-D.** Quantifications of the percentage of NUCB1 or NUCB2 that was packaged into transport vesicles (n = 3, mean ± S.D.). *, $p < 0.05$; **, $p < 0.01$. **E.** Cell lysates from HEK293T cells transfected with N.C. siRNA or siRNA against SURF4 were analyzed by immunoblot. **F.** HEK293T cells expressing SURF4-HA were treated in 2mM DSP, and the cell lysates were incubated with beads conjugated with HA antibodies. After incubation, the bound proteins were eluted analyzed by western blot using the indicated antibodies.
